# Supplementary material for: Generation and application of novel hES cell reporter lines for the differentiation and maturation of hPS cell-derived islet-like clusters
Source: Sci Rep. 2024 Aug 27;14:19863. doi: 10.1038/s41598-024-69645-4 (PMC11350089; doi:10.1038/s41598-024-69645-4)
Supplement: Supplementary file 1 — Supplementary Figures. [file 41598_2024_69645_MOESM1_ESM.pdf]

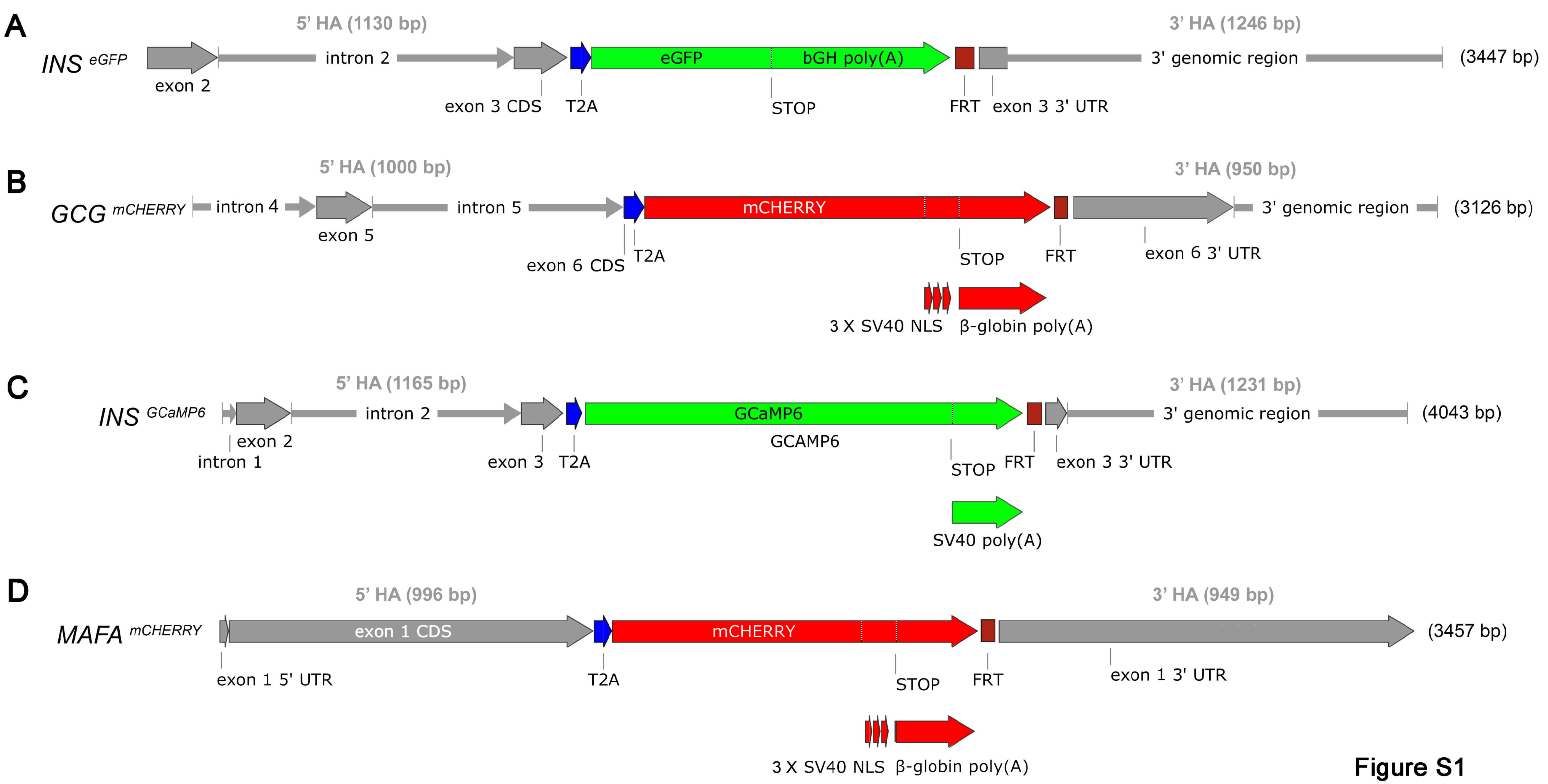
A
5' HA (1130 bp)
3' HA (1246 bp)
INS eGFP
exon 2
intron 2
exon 3 CDS
T2A
eGFP
bGH poly(A)
STOP
FRT
exon 3 3' UTR
3' genomic region
(3447 bp)
B
5' HA (1000 bp)
3' HA (950 bp)
GCG mCHERRY
intron 4
exon 5
intron 5
exon 6 CDS
T2A
mCHERRY
STOP
FRT
exon 6 3' UTR
3' genomic region
(3126 bp)
3 X SV40 NLS
β-globin poly(A)
C
5' HA (1165 bp)
3' HA (1231 bp)
INS GCaMP6
intron 1
exon 2
intron 2
exon 3
T2A
GCaMP6
GCAMP6
STOP
FRT
exon 3 3' UTR
3' genomic region
(4043 bp)
SV40 poly(A)
D
5' HA (996 bp)
3' HA (949 bp)
MAFA mCHERRY
exon 1 5' UTR
exon 1 CDS
T2A
mCHERRY
STOP
FRT
exon 1 3' UTR
(3457 bp)
3 X SV40 NLS
β-globin poly(A)

Figure S1

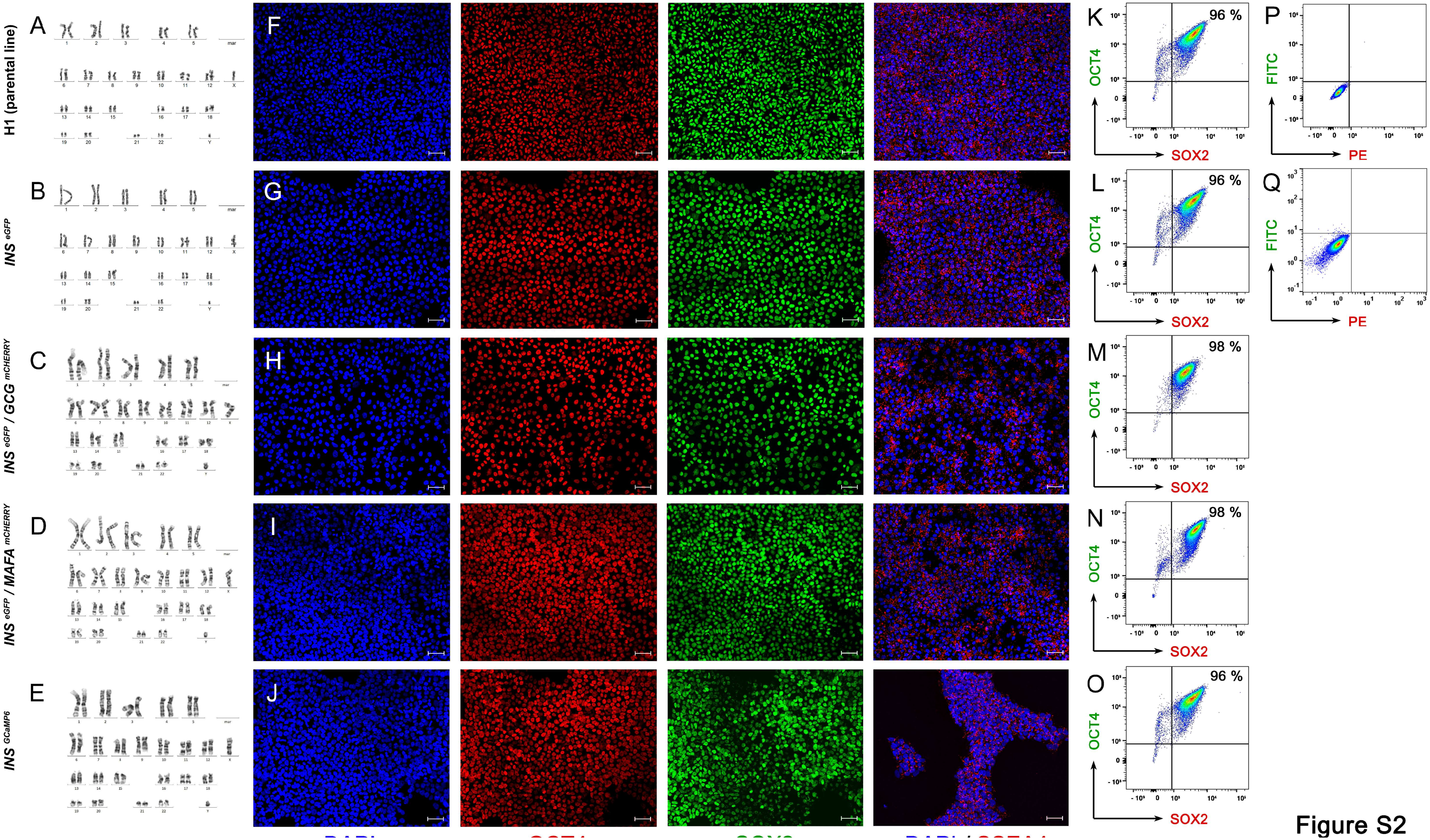
H1 (parental line)
INS eGFP
INS eGFP / GCG mCHERRY
INS eGFP / MAFA mCHERRY
INS GCaMP6
A
B
C
D
E
F
G
H
I
J
K
L
M
N
O
P
Q
96 %
96 %
98 %
98 %
96 %
OCT4
SOX2
FITC
PE

Figure S2

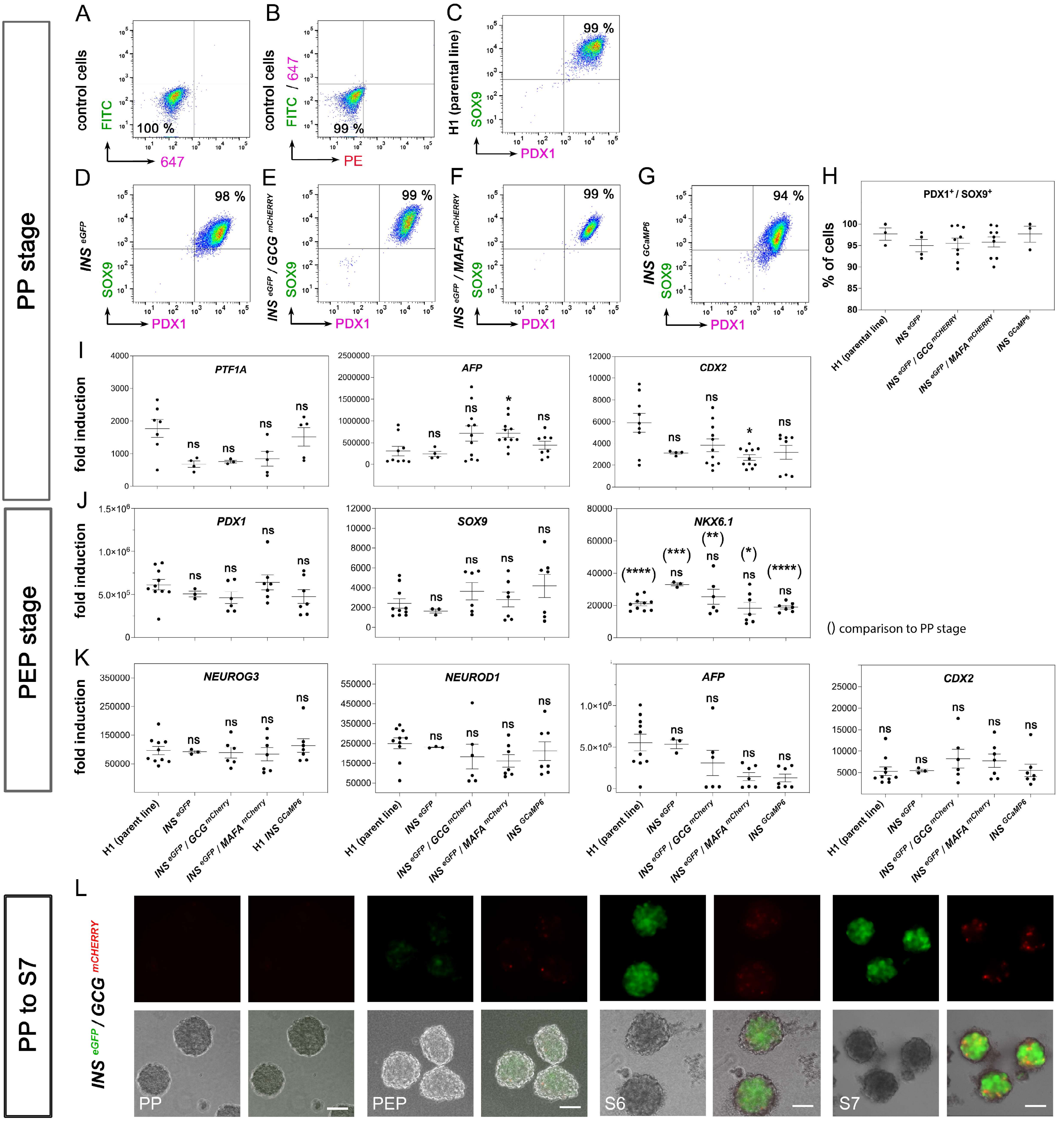
PP stage
PEP stage
PP to S7
A
B
C
D
E
F
G
H
I
J
K
L
control cells
FITC
647
100 %
control cells
FITC / 647
PE
99 %
H1 (parental line)
SOX9
PDX1
99 %
INS eGFP
98 %
INS eGFP / GCG mCHERRY
99 %
INS eGFP / MAFA mCHERRY
99 %
INS GCaMP6
94 %
PDX1+ / SOX9+
% of cells
fold induction
PTF1A
AFP
CDX2
PDX1
SOX9
NKX6.1
ns
() comparison to PP stage
NEUROG3
NEUROD1
H1 (parent line)
INS eGFP
INS eGFP / GCG mCherry
INS eGFP / MAFA mCherry
H1 INS GCaMP6
INS GCaMP6
INS eGFP / GCG mCHERRY
PP
PEP
S6
S7

Figure S3

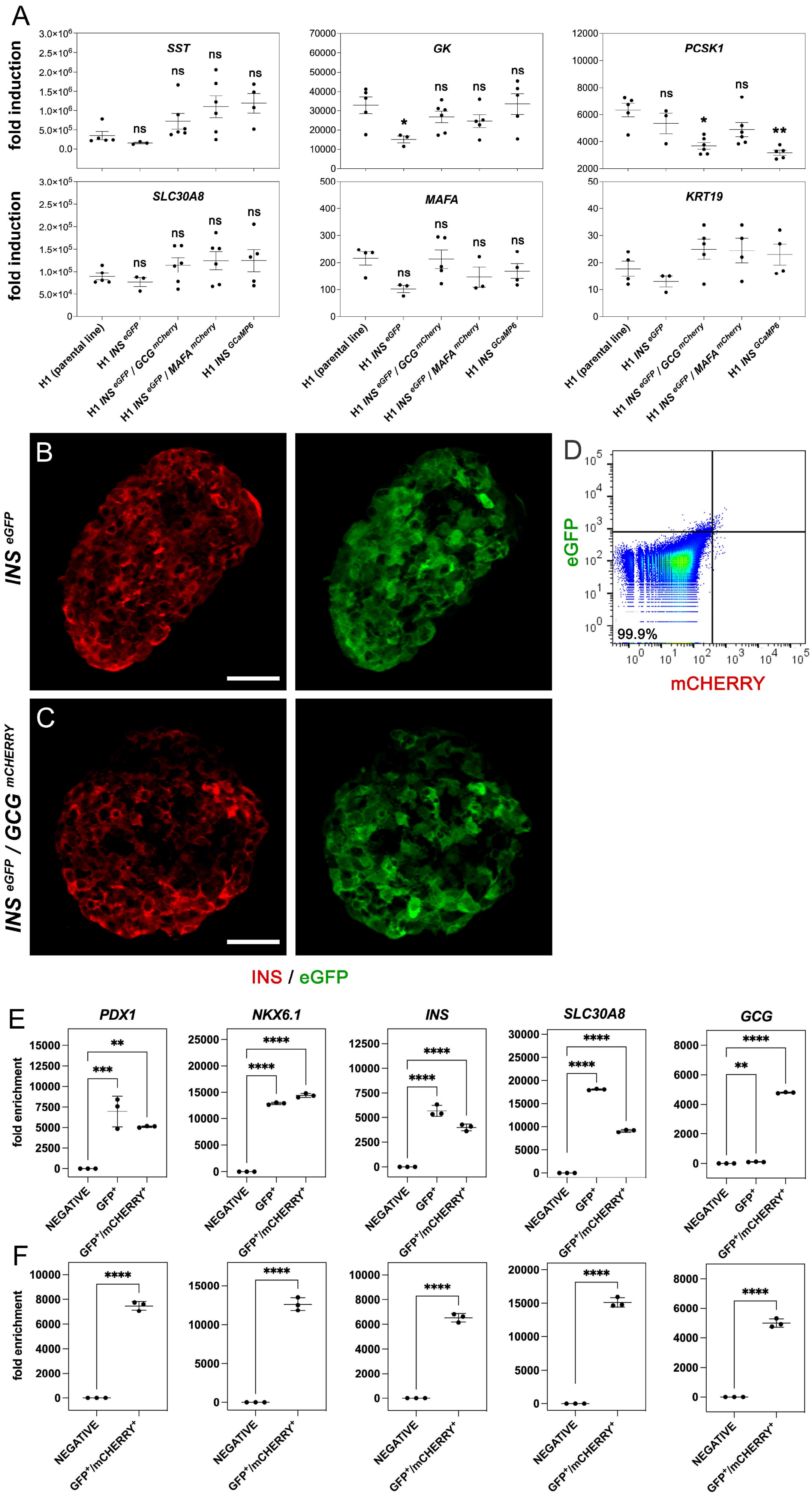

A
SST
GK
PCSK1
SLC30A8
MAFA
KRT19
fold induction
H1 (parental line)
H1 INS eGFP
H1 INS eGFP / GCG mCherry
H1 INS eGFP / MAFA mCherry
H1 INS GCaMP6
B
C
D
INS eGFP
INS eGFP / GCG mCHERRY
eGFP
mCHERRY
99.9%
INS / eGFP
E
F
PDX1
NKX6.1
INS
SLC30A8
GCG
fold enrichment
NEGATIVE
GFP+
GFP+/mCHERRY+
INS eGFP / MAFA mCHERRY

Figure S4

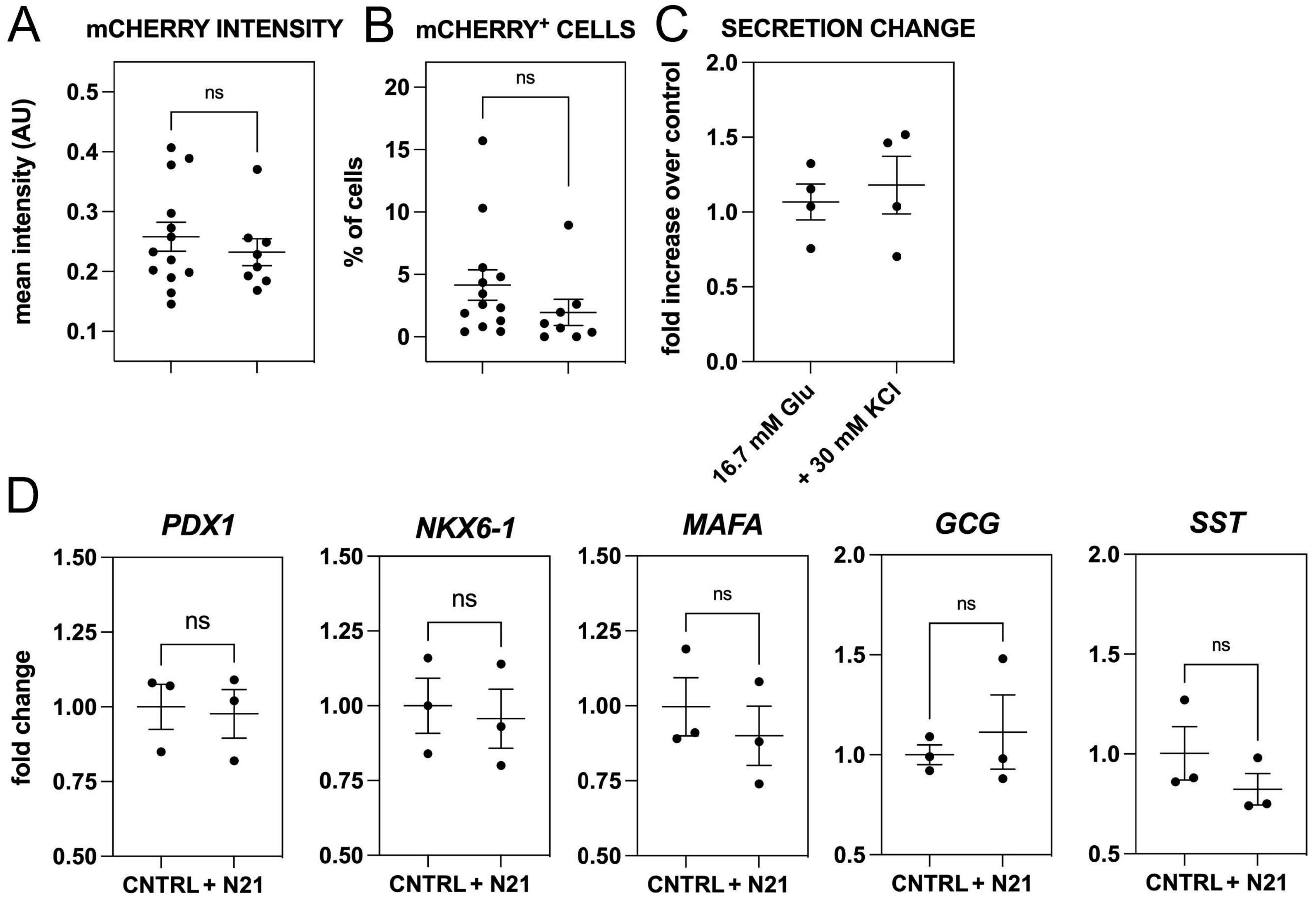
A
mCHERRY INTENSITY
mean intensity (AU)
0.5
0.4
0.3
0.2
0.1
ns
B
mCHERRY+ CELLS
% of cells
20
15
10
5
0
ns
C
SECRETION CHANGE
fold increase over control
2.0
1.5
1.0
0.5
0.0
16.7 mM Glu
+ 30 mM KCl
D
PDX1
NKX6-1
MAFA
GCG
SST
fold change
1.50
1.25
1.00
0.75
0.50
2.0
1.5
1.0
0.5
ns
CNTRL + N21

Figure S5

Figure S1 Detailed maps of the targeted alleles

(A-D) Representation of the loci of the genes of interest after the targeting and the removal of the selection cassette. 5’ and 3’ homology arms (HA) containing introns and coding exons, or 3’ UTRs and downstream genomic regions, respectively, are shown in grey. T2A coding sequences (blue) are inserted in-frame with the amino acid coding part of the last exon of the corresponding gene. The reporter is inserted in-frame with the T2A and is followed by a polyA (red or green). mCherry CDS includes a nuclear localization signal (NLS) at its 3’. The single FRT site left after the Flp recombinase-mediated removal of the selection cassette is shown in purple.

Figure S2 Expression of pluripotency and definitive endoderm markers in H1 cells, derived reporter lines and differentiated cells into definitive endoderm

(A-E) Karyotyping by G-banding for the parental H1 line (A) and in the derived reporter lines H1 *INS* ^{eGFP} (B), H1 *INS* ^{eGFP}/*GCG* ^{mCherry} (C), H1 *INS* ^{eGFP}/*MAFA* ^{mCherry} (D) and H1 *INS* ^{GCaMP6} (E).

(F-J) Fluorescent immunostainings show strong and broad expression of the pluripotent markers OCT4, SOX2 and SSEA4 in the parental H1 (F) and in the derived reporter lines H1 *INS* ^{eGFP} (G), H1 *INS* ^{eGFP}/*GCG* ^{mCherry} (H), H1 *INS* ^{eGFP}/*MAFA* ^{mCherry} (I) and H1 *INS* ^{GCaMP6} (J).

(K-P) Flow Cytometry analyses of H1 (parental line) (K), H1 *INS* ^{eGFP} (L), H1 *INS* ^{eGFP}/*GCG* ^{mCherry} (M), H1 *INS* ^{eGFP}/*MAFA* ^{mCherry} (N) and H1 *INS* ^{GCaMP6} (O) hES cells using antibodies for OCT4 and SOX2. (P) Shows a representative flow cytometry control sample stained only with the secondary antibodies used for the detection of OCT4 and SOX2.

(Q) Representative flow cytometry control sample of DE cells stained only with the secondary antibodies used for the detection of SOX17 and FOXA2.

Scale bar corresponds to 50 µm.

Figure S3 Differentiation efficiency into pancreatic progenitors and pancreatic endocrine progenitors

(A, B) Flow Cytometry analyses of PP cells stained only with the secondary antibodies providing the gating for PDX1^{+}/NKX6.1^{+} cells (A) and the gating for NKX6.1^{+} cells (B).

(C-G) Selected flow cytometry analyses showing the % of double PDX1^{+}/SOX9^{+} cells in H1 (C), H1 *INS* ^{eGFP} (D), H1 *INS* ^{eGFP}/*GCG* ^{mCherry} (E), H1 *INS* ^{eGFP}/*MAFA* ^{mCherry} (F) and H1 *INS* ^{GCaMP6} (G) derived PP cells.

(H) Flow cytometry analyses of PDX1^{+} / SOX9^{+} PP cells derived from the H1 (parental line), H1 *INS* ^{eGFP}, H1 *INS* ^{eGFP}/*GCG* ^{mCherry}, H1 *INS* ^{eGFP}/*MAFA* ^{mCherry} and H1 *INS* ^{GCaMP6} hES cells. Dots represent values from independent experiments and horizontal lines represent the mean ± standard error of the mean (SEM).

(I) Gene expression analyses by RT-qPCR of PP cells, derived from H1 as well as the reporter hPS cell lines, showing the expression of the pancreatic progenitor marker *PTF1A*, the liver marker *AFP* and the gut *CDX2* marker expression. Gene expression levels are shown as fold change relative to those in undifferentiated H1. Dots represent values from independent experiments and horizontal lines represent the mean ± SEM.

(J, K) Gene expression analyses by RT-qPCR of PEP cells, derived from H1 as well as the reporter hPS cell lines, showing the expression of *PDX1*, *SOX9* and *NKX6.1* genes (K), expression of the key endocrine progenitor genes *NEUROG3* and *NEUROD1* in all the lines as well as expression of the liver marker *AFP* and the intestinal marker *CDX2* (L). Gene expression levels are shown as fold change relative to those in undifferentiated H1. Dots represent values from independent experiments and horizontal lines represent the mean ± SEM.

(L) Time course of the *INS* ^{eGFP} and *GCG* ^{mCherry} reporter expression by whole mount live immunofluorescence one day after clustering of the PP cells (PP) and at the end PEP, S6 and S7 stages.

Statistical analyses were performed using the non-parametric Kruskal-Wallis test, using the H1 (parent line) as the basis for the comparison. For the comparison of the *NKX6.1* expression between PEP and PP cells of the same line the Welsh's t-test was used. $p \leq 0.05$ (*), $p \leq 0.005$ (**), $p \leq 0.0005$ (***) and $p \leq 0.0001$ (****). Scale bars correspond to 100 µm

Figure S4 Differentiation efficiency into SC-islets and reporter functionality

(A) Gene expression analyses by RT-qPCR of SC-islets for the hormones *GCG* and *SST*, genes involved in the β-cell maturation and functionality, *PAX4, ZnT8, PC1/3, MAFA* as well as for the hepatic gene marker *AFP,* the gut marker *CDX2* and the duct marker *CK19*. Data are expressed as fold change relative to undifferentiated H1. Dots represent values from independent experiments and horizontal lines represent the mean ± SEM.

(B) Representative immunofluorescent staining of *INS* ^{eGFP} SC-islet cryosections for INS and eGFP.

(C) Representative immunofluorescent staining of *INS* ^{eGFP}/*GCG* ^{mCherry} SC-islet cryosections for INS and eGFP.

(D) Representative flow cytometry analyses of live H1 parental line SC-islet cells used to set the gating for the analyses of the reporter lines.

(E) Fold-enrichment of *PDX1*, *NKX6.1*, *INS*, *GCG* and *ZnT8* expression in GFP^{+} and GFP^{+}/mCHERRY^{+} FACS isolated cells from *INS* ^{eGFP}/*GCG* ^{mCherry} SC-islets. Expression in control cells was set as 1. Dots represent values from independent experiments and horizontal lines represent the mean ± SEM.

(F) Fold-enrichment of *PDX1*, *NKX6.1*, *INS*, *GCG* and *ZnT8* expression in GFP^{+} FACS isolated cells from *INS* ^{eGFP}/*MAFA* ^{mCherry} SC-islets. Expression in control cells was set at 1. Dots represent values from independent experiments and horizontal lines represent the mean ± SEM.

Statistical analyses were performed using the non-parametric Kruskal-Wallis test, using the H1 (parental line) as control for the comparison with $p \leq 0.05$ (*), $p \leq 0.005$ (**) and $p \leq 0.0005$ (***). Scale bars correspond to 50 µm.

Figure S5 N21 promotes β-cell formation and responsiveness

(A, B) Supplementing S7 with N21 did not change the mean mCHERRY intensity per SC-islet (A) or the % of mCHERRY^{+} cells per islet (B) as compared to control (CNTRL) islets. Dots in A, B correspond to individual SC-islets.

(C) Supplementing S7 with N21 did not change the response of SC-islets to either 16.7 mM glucose or to 16.7 mM + 30 mM KCl. Dots represent values from independent differentiation experiments.

(D) Changes in the expression of *PDX-1*, *NKX6-1*, *MAFA*, as well as *GCG* and *SOM* upon supplementation of S7 medium with N21 as compared to control conditions (CNTRL). Dots represent values from independent differentiation experiments.

Statistical analyses were performed using the non-parametric Kruskal-Wallis test, using the H1 (parental line) as control for the comparison with $p \leq 0.05$ (*), $p \leq 0.005$ (**) and $p \leq 0.0005$ (***). Horizontal lines represent the mean ± SEM. Scale bars correspond to 50 µm.
