# Supplementary material for: Generation and application of novel hES cell reporter lines for the differentiation and maturation of hPS cell-derived islet-like clusters
Source: Sci Rep. 2024 Aug 27;14:19863. doi: 10.1038/s41598-024-69645-4 (PMC11350089; doi:10.1038/s41598-024-69645-4)
Supplement: Supplementary file 2 — Supplementary Information. [file 41598_2024_69645_MOESM2_ESM.pdf]

Sequence: SUP FILE 1 INS eGFP REPORTER copy.dna (Circular / 3446 bp)
Features: 41 total

5′ gccatggccctgtggatgcgcctcctgcccctgctggcgctgctggccctctggggacctgaccc 65
3′ cggtaccgggacacctacgcggaggacggggacgaccgcgacgaccgggagacccctggactggg

1 Ala Met Ala Leu 5 Trp Met Arg Leu Leu 10 Pro Leu Leu Ala Leu 15 Leu Ala Leu Trp Gly 20 Pro Asp Pro

exon 2

Met Ala Leu Trp Met Arg Leu Leu Pro Leu Leu Ala Leu Leu Ala Leu Trp Gly Pro Asp Pro

Trp Pro Gly Thr Ser Ala Gly Gly Ala Gly Ala Pro Ala Ala Pro Gly Arg Pro Val Gln Gly

agccgcagcctttgtgaaccaacacctgtgcggctcacacctggtggaagctctctacctagtgt 130
tcggcgtcggaaacacttggttgtggacacgccgagtgtggaccaccttcgagagatggatcaca

Ala Ala 25 Ala Phe Val Asn Gln 30 His Leu Cys Gly Ser 35 His Leu Val Glu Ala 40 Leu Tyr Leu Val

exon 2

Ala Ala Ala Phe Val Asn Gln His Leu Cys Gly Ser His Leu Val Glu Ala Leu Tyr Leu Val

Leu Arg Leu Arg Gln Ser Gly Val Gly Thr Arg Ser Val Gly Pro Pro Leu Glu Arg Gly Leu Thr

gcggggaacgaggcttcttctacacacccaagacccgccgggaggcagaggacctgcagggtgag 195
cgccccttgctccgaagaagatgtgtgggttctgggcggccctccgtctcctggacgtcccactc

intron 2

Cys 45 Gly Glu Arg Gly Phe 50 Phe Tyr Thr Pro Lys 55 Thr Arg Arg Glu Ala 60 Glu Asp Leu Gln Gly Glu

exon 2

Cys Gly Glu Arg Gly Phe Phe Tyr Thr Pro Lys Thr Arg Arg Glu Ala Glu Asp Leu Gln Gly Glu

Arg Pro Val Leu Ser Arg Arg Cys Val Trp Ser Gly Gly Pro Pro Leu Pro Gly Ala Pro His Ala

ccaactgcccattgctgcccctggccgcccccagccacccccctgctcctggcgctcccacccagc 260
ggttgacgggtaacgacggggaccggcgggggtcggtgggggacgaggaccgcgagggtgggtcg

intron 2

Pro Thr Ala His Cys Cys Pro Trp Pro Pro Pro Ala Thr Pro Cys Ser Trp Arg Ser His Pro Ala
(in frame with exon 2)

Pro Thr Ala His Cys Cys Pro Trp Pro Pro Pro Ala Thr Pro Cys Ser Trp Arg Ser His Pro Ala

Leu Gln Gly Asn Ser Gly Arg Ala Ala Gly Leu Trp Gly Arg Ser Arg Ala Ser Gly Gly Leu

atgggcagaagggggcaggaggctgccacccagcaggggggtcaggtgcactttttaaaaagaag 325
tacccgtcttcccccgtcctccgacggtgggtcgtccccccagtccacgtgaaaaatttttcttc

intron 2

Trp Ala Glu Gly Gly Arg Arg Leu Pro Pro Ser Arg Gly Ser Gly Ala Leu Phe *
(in frame with exon 2)

Trp Ala Glu Gly Gly Arg Arg Leu Pro Pro Ser Arg Gly Ser Gly Ala Leu Phe *

Met Gly Arg Arg Gly Gln Glu Ala Ala Thr Gln Gln Gly Val Arg Cys Thr Phe Leu Lys Arg Ser

Met

ttctcttggtcacgtcctaaaagtgaccagctccctgtggcccagtcagaatctcagcctgagga 390
aagagaaccagtgcaggattttcactggtcgagggacaccgggtcagtcttagagtcggactcct

intron 2

Ser Leu Gly His Val Leu Lys Val Thr Ser Ser Leu Trp Pro Ser Gln Asn Leu Ser Leu Arg

cggtgttggcttcggcagccccgagatacatcagagggtgggcacgctcctccctccactcgccc 455
gccacaaccgaagccgtcggggctctatgtagtctcccacccgtgcgaggagggaggtgagcggg
intron 2
Thr Val Leu Ala Ser Ala Ala Pro Arg Tyr Ile Arg Gly Trp Ala Arg Ser Ser Leu His Ser Pro

ctcaaacaaatgccccgcagcccatttctccaccctcatttgatgaccgcagattcaagtgtttt 520
gagtttgtttacggggcgtcgggtaaagaggtgggagtaaactactggcgtctaagttcacaaaa
intron 2
Leu Lys Gln Met Pro Arg Ser Pro Phe Leu His Pro His Leu Met Thr Ala Asp Ser Ser Val Leu

gttaagtaaagtcctgggtgacctggggtcacagggtgccccacgctgcctgcctctgggcgaac 585
caattcatttcaggacccactggaccccagtgtcccacggggtgcgacggacggagacccgcttg
intron 2
Leu Ser Lys Val Leu Gly Asp Leu Gly Ser Gln Gly Ala Pro Arg Cys Leu Pro Leu Gly Glu

accccatcacgcccggaggagggcgtggctgcctgcctgagtgggccagacccctgtcgccaggc 650
tggggtagtgcgggcctcctcccgcaccgacggacggactcacccggtctggggacagcggtccg
intron 2
His Pro Ile Thr Pro Gly Gly Gly Arg Gly Cys Leu Pro Glu Trp Ala Arg Pro Leu Ser Pro Gly

ctcacggcagctccatagtcaggagatggggaagatgctggggacaggccctggggagaagtact 715
gagtgccgtcgaggtatcagtcctctaccccttctacgacccctgtccgggacccctcttcatga
intron 2
Leu Thr Ala Ala Pro *

gggatcacctgttcaggctcccactgtgacgctgccccggggcgggggaaggaggtgggacatgt 780
ccctagtggacaagtccgagggtgacactgcgacggggccccgcccccttcctccaccctgtaca
intron 2

gggcgttggggcctgtaggtccacacccagtgtgggtgaccctccctctaacctgggtccagccc 845
cccgcaaccccggacatccaggtgtgggtcacacccactgggagggagattggacccaggtcggg
intron 2
* Gly Pro Asp Leu Gly

ggctggagatgggtgggagtgcgacctagggctggcgggcaggcgggcactgtgtctccctgact 910
ccgacctctacccaccctcacgctggatcccgaccgcccgtccgcccgtgacacagagggactga
intron 2
Ala Pro Ser Pro His Ser His Ser Arg Pro Ser Ala Pro Leu Arg Ala Ser His Arg Gly Ser Gln

gtgtcctcctgtgtccctctgcctcgccgctgttccggaacctgctctgcgcggcacgtcctggc 975
cacaggaggacacagggagacggagcggcgacaaggccttggacgagacgcgccgtgcaggaccg

intron 2

Thr Arg Arg His Gly Glu Ala Glu Gly Ser Asn Arg Phe Arg Ser Gln Ala Ala Arg Gly Pro

agtggggcaggtggagctgggcgggggccctggtgcaggcagcctgcagcccttggccctggagg 1040
tcaccccgtccacctcgacccgcccccgggaccacgtccgtcggacgtcgggaaccgggacctcc

exon 3 CDS
intron 2

Leu Pro Ala Pro Pro Ala Pro Arg Pro Gly Gln His Leu Cys Gly Ala Ala Arg Pro Gly Pro Pro

ggtccctgcagaagcgtggcattgtggaacaatgctgtacAagcatctgctccctctaccagctg 1105
ccagggacgtcttcgcaccgtaacaccttgttacgacatgTtcgtagacgagggagatggtcgac

exon 3 CDS

Thr Gly Ala Ser Ala His Cys Gln Pro Val Ile Ser Tyr Leu Cys Arg Ser Gly Arg Gly Ala Pro

gagaactactgcaacGGCAGTGGAGAGGGCAGAGGAAGTCTGCTAACATGCGGTGACGTCGAGGA 1170
ctcttgatgacgttgCCGTCACCTCTCCCGTCTCCTTCAGACGATTGTACGCCACTGCAGCTCCT

140 145 150
Glu Gly Arg Gly Ser Leu Leu Thr Cys Gly Asp Val Glu Glu

exon 3 CDS
T2A

Ser Ser Ser Cys Arg Cys His Leu Pro Cys Leu Phe Asp Ala Leu Met

GAATCCTGGCCCAATGAGCAAGGGCGAGGAGCTGTTCACCGGGGTGGTGCCCATCCTGGTCGAGC 1235
CTTAGGACCGGGTTACTCGTTCCCGCTCCTCGACAAGTGGCCCCACCACGGGTAGGACCAGCTCG

155
Asn Pro Gly Pro
5 10 15
Met Ser Lys Gly Glu Glu Leu Phe Thr Gly Val Val Pro Ile Leu Val Glu

eGFP
T2A
eGFP

Met Ser Lys Gly Glu Glu Leu Phe Thr Gly Val Val Pro Ile Leu Val Glu

TGGACGGCGACGTAAACGGCCACAAGTTCAGCGTGTCCGGCGAGGGCGAGGGCGATGCCACCTAC 1300
ACCTGCCGCTGCATTTGCCGGTGTTCAAGTCGCACAGGCCGCTCCCGCTCCCGCTACGGTGGATG

20 25 30 35
Leu Asp Gly Asp Val Asn Gly His Lys Phe Ser Val Ser Gly Glu Gly Glu Gly Asp Ala Thr Tyr

eGFP
eGFP

Leu Asp Gly Asp Val Asn Gly His Lys Phe Ser Val Ser Gly Glu Gly Glu Gly Asp Ala Thr Tyr

GGCAAGCTGACCCTGAAGTTCATCTGCACCACCGGCAAGCTGCCCGTGCCCTGGCCCACCCTCGT 1365
CCGTTCGACTGGGACTTCAAGTAGACGTGGTGGCCGTTCGACGGGCACGGGACCGGGTGGGAGCA

40 45 50 55 60
Gly Lys Leu Thr Leu Lys Phe Ile Cys Thr Thr Gly Lys Leu Pro Val Pro Trp Pro Thr Leu Val

eGFP
eGFP

Gly Lys Leu Thr Leu Lys Phe Ile Cys Thr Thr Gly Lys Leu Pro Val Pro Trp Pro Thr Leu Val

```
GACCACCCTGACCTACGGCGTGCAGTGCTTCAGCCGCTACCCCGACCACATGAAGCAGCACGACT   1430
CTGGTGGGACTGGATGCCGCACGTCACGAAGTCGGCGATGGGGCTGGTGTACTTCGTCGTGCTGA
          65                 70                 75                 80
Thr Thr Leu Thr Tyr Gly Val Gln Cys Phe Ser Arg Tyr Pro Asp His Met Lys Gln His Asp
eGFP >
eGFP
Thr Thr Leu Thr Tyr Gly Val Gln Cys Phe Ser Arg Tyr Pro Asp His Met Lys Gln His Asp

TCTTCAAGTCCGCCATGCCCGAAGGCTACGTCCAGGAGCGCACCATCTTCTTCAAGGACGACGGC   1495
AGAAGTTCAGGCGGTACGGGCTTCCGATGCAGGTCCTCGCGTGGTAGAAGAAGTTCCTGCTGCCG
      85                 90                 95                 100
Phe Phe Lys Ser Ala Met Pro Glu Gly Tyr Val Gln Glu Arg Thr Ile Phe Phe Lys Asp Asp Gly
eGFP >
eGFP
Phe Phe Lys Ser Ala Met Pro Glu Gly Tyr Val Gln Glu Arg Thr Ile Phe Phe Lys Asp Asp Gly

AACTACAAGACCCGCGCCGAGGTGAAGTTCGAGGGCGACACCCTGGTGAACCGCATCGAGCTGAA   1560
TTGATGTTCTGGGCGCGGCTCCACTTCAAGCTCCCGCTGTGGGACCACTTGGCGTAGCTCGACTT
105                 110                 115                 120                 125
Asn Tyr Lys Thr Arg Ala Glu Val Lys Phe Glu Gly Asp Thr Leu Val Asn Arg Ile Glu Leu Lys
eGFP >
eGFP
Asn Tyr Lys Thr Arg Ala Glu Val Lys Phe Glu Gly Asp Thr Leu Val Asn Arg Ile Glu Leu Lys

GGGCATCGACTTCAAGGAGGACGGCAACATCCTGGGGCACAAGCTGGAGTACAACTACAACAGCC   1625
CCCGTAGCTGAAGTTCCTCCTGCCGTTGTAGGACCCCGTGTTCGACCTCATGTTGATGTTGTCGG
              130                 135                 140                 145
Gly Ile Asp Phe Lys Glu Asp Gly Asn Ile Leu Gly His Lys Leu Glu Tyr Asn Tyr Asn Ser
eGFP >
eGFP
Gly Ile Asp Phe Lys Glu Asp Gly Asn Ile Leu Gly His Lys Leu Glu Tyr Asn Tyr Asn Ser

ACAACGTCTATATCATGGCCGACAAGCAGAAGAACGGCATCAAGTGAACTTCAAGATCCGCCACA   1690
TGTTGCAGATATAGTACCGGCTGTTCGTCTTCTTGCCGTAGTTCACTTGAAGTTCTAGGCGGTGT
        150                 155                            165                 170
His Asn Val Tyr Ile Met Ala Asp Lys Gln Lys Asn Gly Ile Lys * Thr Ser Arg Ser Ala Thr
eGFP | bGH poly(A) >
eGFP
His Asn Val Tyr Ile Met Ala Asp Lys Gln Lys Asn Gly Ile Lys *

ACATCGAGGACGGCAGCGTGCAGCTCGCCGACCACTACCAGCAGAACACCCCCATCGGCGACGGC   1755
TGTAGCTCCTGCCGTCGCACGTCGAGCGGCTGGTGATGGTCGTCTTGTGGGGGTAGCCGCTGCCG
                 175                 180                 185                 190
Thr Ser Arg Thr Ala Ala Cys Ser Ser Pro Thr Thr Thr Ser Arg Thr Pro Pro Ser Ala Thr Ala
bGH poly(A) >
eGFP

CCCGTGCTGCTGCCCGACAACCACTACCTGAGCACCCAGTCCGCCCTGAGCAAAGACCCCAACGA   1820
GGGCACGACGACGGGCTGTTGGTGATGGACTCGTGGGTCAGGCGGGACTCGTTTCTGGGGTTGCT
        195                 200                 205                 210
Pro Cys Cys Cys Pro Thr Thr Thr Thr * Ala Pro Ser Pro Pro * Ala Lys Thr Pro Thr
bGH poly(A) >
eGFP
```

GAAGCGCGATCACATGGTCCTGCTGGAGTTCGTGACCGCCGCCGGGATCACTCTCGGCATGGACG 1885
CTTCGCGCTAGTGTACCAGGACGACCTCAAGCACTGGCGGCGGCCCTAGTGAGAGCCGTACCTGC
215 220 225 230 235
Arg Ser Ala Ile Thr Trp Ser Cys Trp Ser Ser * Pro Pro Pro Gly Ser Leu Ser Ala Trp Thr
bGH poly(A)
eGFP

AGCTGTACAAGgaattctaaCTAGAGCTCGCTGATCAGCCTCGACTGTGCCTTCTAGTTGCCAGC 1950
TCGACATGTTCcttaagattGATCTCGAGCGACTAGTCGGAGCTGACACGGAAGATCAACGGTCG
240 245 250 255
Ser Cys Thr Arg Asn Ser Asn * Ser Ser Leu Ile Ser Leu Asp Cys Ala Phe * Leu Pro Ala
bGH poly(A)
eGFP
* Gly Arg Ser His Arg Arg Thr Ala Leu

CATCTGTTGTTTGCCCCTCCCCCGTGCCTTCCTTGACCCTGGAAGGTGCCACTCCCACTGTCCTT 2015
GTAGACAACAAACGGGGAGGGGGCACGGAAGGAACTGGGACCTTCCACGGTGAGGGTGACAGGAA
260 265 270 275
Ile Cys Cys Leu Pro Leu Pro Arg Ala Phe Leu Asp Pro Gly Arg Cys His Ser His Cys Pro
bGH poly(A)
eGFP
Trp Arg Asn Asn Ala Gly Gly Gly His Arg Gly Gln Gly Gln Phe Thr Gly Ser Gly Ser Asp Lys

TCCTAATAAAATGAGGAAATTGCATCGCATTGTCTGAGTAGGTGTCATTCTATTCTGGGGGGTGG 2080
AGGATTATTTTACTCCTTTAACGTAGCGTAACAGACTCATCCACAGTAAGATAAGACCCCCCACC
280 285 290 295 300
Phe Leu Ile Lys * Gly Asn Cys Ile Ala Leu Ser Glu * Val Ser Phe Tyr Ser Gly Gly Trp
bGH poly(A)
eGFP
Gly Leu Leu Ile Leu Phe Asn Cys Arg Met Thr Gln Thr Pro Thr Met Arg Asn Gln Pro Thr Pro

GGTGGGGCAGGACAGCAAGGGGGAGGATTGGGAAGAgAATAGCAGGCATGCTGGGGAgcgCTAGA 2145
CCACCCCGTCCTGTCGTTCCCCCTCCTAACCCTTCTcTTATCGTCCGTACGACCCCTcgcGATCT
305 310 315
Gly Gly Ala Gly Gln Gln Gly Gly Gly Leu Gly Arg Glu * Gln Ala Cys Trp Gly Ala Leu Glu
bGH poly(A)
eGFP
His Pro Leu Val Ala Leu Pro Leu Ile Pro Phe Leu Ile Ala Pro Met Ser Pro Leu Ala Leu

GTCAACGAAGTTCCTATACTTTCTAGAGAATAGGAACTTCGGAATAGGAACTTCGTTGACCTAGA 2210
CAGTTGCTTCAAGGATATGAAAGATCTCTTATCCTTGAAGCCTTATCCTTGAAGCAACTGGATCT
Ser Thr Lys Phe Leu Tyr Phe Leu Glu Asn Arg Asn Phe Gly Ile Gly Thr Ser Leu Thr *
(in frame with eGFP)
FRT
Thr Leu Ser Thr Gly Ile Ser Glu Leu Ser Tyr Ser Ser Arg Phe Leu Phe Lys Thr Ser Arg Ser

GTCGacgcagcccgcagAcagccccacacccgccgcctcctgcaccgagagagatggaataaagc 2275
CAGCtgcgtcgggcgtcTgtcggggtgtgggcggcggaggacgtggctctctctaccttatttcg
exon 3 3' UTR
Asp Val Cys Gly Ala Ser Leu Gly Val Gly Ala Ala Glu Gln Val Ser Leu Ser Pro Ile Phe Gly

ccttgaaccagccctgctgtgccgtctgtgtgtcttgggggccctgggccaagccccacttcccg 2340
ggaacttggtcgggacgacacggcagacacacagaacccccgggacccggttcggggtgaagggc

exon 3 3' UTR — 3' genomic region

Lys Phe Trp Gly Gln Gln Ala Thr Gln Thr Asp Gln Pro Gly Gln Ala Leu Gly Trp Lys Gly

gcactgttgtgagcccctcccagctctctccacgctctctgggtgcccacaggtgccaacgccgg 2405
cgtgacaacactcggggagggtcgagagaggtgcgagagacccacgggtgtccacggttgcggcc

3' genomic region

Ala Ser Asn His Ala Gly Gly Leu Glu Arg Trp Ala Arg Gln Thr Gly Val Pro Ala Leu Ala Pro

ccaggcccagcatgcagtggctctccccaaagcggccatgcctgtcggctgcctgctgcccccac 2470
ggtccgggtcgtacgtcaccgagaggggtttcgccggtacggacagccgacggacgacgggggtg

3' genomic region

Trp Ala Trp Cys Ala Thr Ala Arg Gly Leu Ala Ala Met Gly Thr Pro Gln Arg Ser Gly Gly Gly

cctgtggctcagggtccagtatgggagctgcgggggtctctgaggggccaggggtggtggggcca 2535
ggacaccgagtcccaggtcataccctcgacgcccccagagactccccggtccccaccaccccggt

3' genomic region

Gln Pro Glu Pro Asp Leu Ile Pro Ala Ala Pro Thr Glu Ser Pro Gly Pro Thr Thr Pro Gly

ctgagaagtgacttcttgttcagtagctctggactcttggagtccccagagaccttgttcaggaa 2600
gactcttcactgaagaacaagtcatcgagacctgagaacctcaggggtctctggaacaagtcctt

3' genomic region

Ser Leu Leu Ser Lys Lys Asn Leu Leu Glu Pro Ser Lys Ser Asp Gly Ser Val Lys Asn Leu Phe

agggaatgagaacattccagcaattttcccccacctagccctcccaggttctatttttagagtt 2665
tcccttactcttgtaaggtcgttaaaagggggtggatcgggagggtccaagataaaaatctcaa

3' genomic region

Met Arg Thr Phe Gln Gln Phe Ser Pro His Leu Ala Leu Pro Gly Ser Ile Phe Arg Val

Pro Phe Ser Phe Met

atttctgatggagtccctgtggagggaggaggctgggctgagggagggggtcctgcagggcgggg 2730
taaagactacctcagggacacctccctcctccgacccgactccctcccccaggacgtcccgcccc

3' genomic region

Ile Ser Asp Gly Val Pro Val Glu Gly Gly Gly Trp Ala Glu Gly Gly Gly Pro Ala Gly Arg Gly

ggctgggaaggtggggagaggctgccgagagccacccgctatccccagctctgggcagccccggg 2795
ccgacccttccacccctctccgacggctctcggtgggcgataggggtcgagacccgtcggggccc

3' genomic region

Ala Gly Lys Val Gly Arg Gly Cys Arg Glu Pro Pro Ala Ile Pro Ser Ser Gly Gln Pro Arg

acagtcacacaccctggcctcgcggcccaagctggcagccgtctgcagccacagcttatgccagc 2860
tgtcagtgtgtgggaccggagcgccgggttcgaccgtcggcagacgtcggtgtcgaatacggtcg
3' genomic region
Asp Ser His Thr Pro Trp Pro Arg Gly Pro Ser Trp Gln Pro Ser Ala Ala Thr Ala Tyr Ala Ser

ccaggtccagccagacacctgagggacccactggtgccttggaggaagcaggagaggtcagatgg 2925
ggtccaggtcggtctgtggactccctgggtgaccacggaacctccttcgtcctctccagtctacc
3' genomic region
Pro Gly Pro Ala Arg His Leu Arg Asp Pro Leu Val Pro Trp Arg Lys Gln Glu Arg Ser Asp Gly

caccatgagctggggcaggtgcagggaccgtggcagcacctggcagggcctcagaacccatgcct 2990
gtggtactcgaccccgtccacgtccctggcaccgtcgtggaccgtcccggagtcttgggtacgga
3' genomic region
Thr Met Ser Trp Gly Arg Cys Arg Asp Arg Gly Ser Thr Trp Gln Gly Leu Arg Thr His Ala

tgggcaccccggccatgaggccctgaggattgcagcccaggagaagcagggaaccgccagggcca 3055
acccgtggggccggtactccgggactcctaacgtcgggtcctcttcgtcccttggcggtcccggt
3' genomic region
Leu Gly Thr Pro Ala Met Arg Pro *

caggggcagagaccagggccagggtccccctgcagccccttagcccaccccctcccagtaagcag 3120
gtccccgtctctggtcccggtcccagggggacgtcggggaatcgggtgggggagggtcattcgtc
3' genomic region

ggctgcttggctggcttcctttgctacagacctgctgctcacccagaagggcccacgggccctgg 3185
ccgacgaaccgaccgaaggaaacgatgtctggacgacgagtgggtcttcccgggtgcccgggacc
3' genomic region

tgacaaggtcgttgtggctccaggtccttggggggtcctgacacagagcctcttctgcagcacccc 3250
actgttccagcaacaccgaggtccaggaacccccaggactgtgtctcggagaagacgtcgtgggg
3' genomic region

tgaggacagggtggctccgctgggcacccagcctagtgggcagacgagaacctaggggctgcctg 3315
actcctgtcccaccgaggcgacccgtgggtcggatcacccgtctgctcttggatccccgacggac
3' genomic region

ggcctactgtggcctgggaggtcagcgggtgaccctagctaccctgtggctgggccagtctgcct 3380
ccggatgacaccggaccctccagtcgcccactgggatcgatgggacaccgacccggtcagacgga
3' genomic region

```
gccacccaggccaaaccaatGCGGCCGCCATGGTCATAGCTGTTTGACGTCAGGTGGCACTTTTC    3445
cggtgggtccggtttggttaCGCCGGCGGTACCAGTATCGACAAACTGCAGTCCACCGTGAAAAG
                     3' genomic region
                                                   * Thr Ala Ser Lys

G      3'
  •••      3446
C      5'
3' genomic region
Pro
```

```
5′ cagctctgctactttctctttccagtagtcactggtccacatgggttttatcaggtatttcaca 65
3′ gtcgagacgatgaaagagaaaggtcatcagtgaccaggtgtacccaaaatagtccataaagtgt
   intron 4

gtacaccctttaaaaacagaatcctttactgtttcctcaagacacttgtgcatgttaccagtggt 130
catgtgggaaatttttgtcttaggaaatgacaaaggagttctgtgaacacgtacaatggtcacca
intron 4

agacaatctgtgatcatttattgaaaatatctaatcaaacgctaattttaacacttatttctta 195
tctgttagacactagtaaataacttttatagattagtttgcgattaaaattgtgaataaaagaat
intron 4

gtttcccagaagaggtcgccattgttgaagaacttggccgcagacatgctgatggttctttctct 260
caaagggtcttctccagcggtaacaacttcttgaaccggcgtctgtacgactaccaagaaagaga
                  135                     140                     145                     150
Phe Pro Glu Glu Val Ala Ile Val Glu Glu Leu Gly Arg Arg His Ala Asp Gly Ser Phe Ser
exon 5
intron 4

gatgagatgaacaccattcttgataatcttgccgccagggactttataaactggttgattcagac 325
ctactctacttgtggtaagaactattagaacggcggtccctgaaatatttgaccaactaagtctg
          155                     160                     165                     170
Asp Glu Met Asn Thr Ile Leu Asp Asn Leu Ala Ala Arg Asp Phe Ile Asn Trp Leu Ile Gln Thr
exon 5

caaaatcactgacaggtgactgcttttttagttaattctgaaaaccatcaaattctcataagtact 390
gttttagtgactgtccactgacgaaaaatcaattaagacttttggtagtttaagagtattcatga
intron 5
 175
Lys Ile Thr Asp Arg *
exon 5

gattgtcatactgctgcaagctgttccccatgtagggggaaagtctataatctcttattatatag 455
ctaacagtatgacgacgttcgacaaggggtacatccccctttcagatattagagaataatatatc
intron 5

gaaataattatgcatgtttaagtcatataagagtacatcattatgtttgttttgtatcattacag 520
ctttattaatacgtacaaattcagtatattctcatgtagtaatacaaacaaaacatagtaatgtc
intron 5

tttgtatctatttattcatttattccagaaacatttactgagtgtctattagagtcaagaagcta 585
aaacatagataaataagtaaataaggtctttgtaaatgactcacagataatctcagttcttcgat
intron 5
```

```
gaaacacagatagaaatgaaacatgggaaatgttacatcattcacatattgtcacattaaaaatt   650
ctttgtgtctatctttactttgtaccctttacaatgtagtaagtgtataacagtgtaatttttaa
intron 5

gctatgttgaaatttcagcacaaaggtgaaatgcagcatatacaatattacatacttaatttaat   715
cgatacaactttaaagtcgtgtttccactttacgtcgtatatgttataatgtatgaattaaatta
intron 5

aagaagagtagtgagaactggacaccgaaaaatacttatcttgatgaattttgattttttaataa   780
ttcttctcatcactcttgacctgtggctttttatgaatagaactacttaaaactaaaaaattatt
intron 5

ttattcataattgtttgatagcattgatgagcagactttaggataaataatctttaaatgaaaat   845
aataagtattaacaaactatcgtaactactcgtctgaaatcctatttattagaaatttactttta
intron 5

atttaagagtccaaaggatggagggatttatggacaaagggattaataaacaacttttatcaaa   910
taaaattctcaggtttcctacctccctaaatacctgtttccctaattatttgttgaaaatagttt
intron 5

ataaatcattgaaatattttctggataagttaatgatatcatcttattataattatgttaaatca   975
tatttagtaactttataaaagacctattcaattactatagtagaataatattaatacaatttagt
intron 5

ttttcttttttaatctctaggaaagagggcagaggaagtcttctaacatgcggtgacgtggagg   1040
aaaagaaaaaaattagagatcctttctcccgtctccttcagaagattgtacgccactgcacctcc
intron 5   T2A
exon 6 CDS

agaatcccggccctATGGTGAGCAAGGGCGAGGAGGATAACATGGCCATCATCAAGGAGTTCATG   1105
tcttagggccgggaTACCACTCGTTCCCGCTCCTCCTATTGTACCGGTAGTAGTTCCTCAAGTAC
mCHERRY
T2A

CGCTTCAAGGTGCACATGGAGGGCTCCGTGAACGGCCACGAGTTCGAGATCGAGGGCGAGGGCGA   1170
GCGAAGTTCCACGTGTACCTCCCGAGGCACTTGCCGGTGCTCAAGCTCTAGCTCCCGCTCCCGCT
mCHERRY

GGGCCGCCCCTACGAGGGCACCCAGACCGCCAAGCTGAAGGTGACCAAGGGTGGCCCCCTGCCCT   1235
CCCGGCGGGGATGCTCCCGTGGGTCTGGCGGTTCGACTTCCACTGGTTCCCACCGGGGGACGGGA
mCHERRY
```

```
TCGCCTGGGACATCCTGTCCCCTCAGTTCATGTACGGCTCCAAGGCCTACGTGAAGCACCCCGCC   1300
AGCGGACCCTGTAGGACAGGGGAGTCAAGTACATGCCGAGGTTCCGGATGCACTTCGTGGGGCGG
mCHERRY

GACATCCCCGACTACTTGAAGCTGTCCTTCCCCGAGGGCTTCAAGTGGGAGCGCGTGATGAACTT   1365
CTGTAGGGGCTGATGAACTTCGACAGGAAGGGGCTCCCGAAGTTCACCCTCGCGCACTACTTGAA
mCHERRY

CGAGGACGGCGGCGTGGTGACCGTGACCCAGGACTCCTCCCTGCAGGACGGCGAGTTCATCTACA   1430
GCTCCTGCCGCCGCACCACTGGCACTGGGTCCTGAGGAGGGACGTCCTGCCGCTCAAGTAGATGT
mCHERRY

AGGTGAAGCTGCGCGGCACCAACTTCCCCTCCGACGGCCCCGTAATGCAGAAGAAGACCATGGGC   1495
TCCACTTCGACGCGCCGTGGTTGAAGGGGAGGCTGCCGGGGCATTACGTCTTCTTCTGGTACCCG
mCHERRY

TGGGAGGCCTCCTCCGAGCGGATGTACCCCGAGGACGGCGCCCTGAAGGGCGAGATCAAGCAGAG   1560
ACCCTCCGGAGGAGGCTCGCCTACATGGGGCTCCTGCCGCGGGACTTCCCGCTCTAGTTCGTCTC
mCHERRY

GCTGAAGCTGAAGGACGGCGGCCACTACGACGCTGAGGTCAAGACCACCTACAAGGCCAAGAAGC   1625
CGACTTCGACTTCCTGCCGCCGGTGATGCTGCGACTCCAGTTCTGGTGGATGTTCCGGTTCTTCG
mCHERRY

CCGTGCAGCTGCCCGGCGCCTACAACGTCAACATCAAGTTGGACATCACCTCCCACAACGAGGAC   1690
GGCACGTCGACGGGCCGCGGATGTTGCAGTTGTAGTTCAACCTGTAGTGGAGGGTGTTGCTCCTG
mCHERRY

TACACCATCGTGGAACAGTACGAACGCGCCGAGGGCCGCCACTCCACCGGCGGCATGGACGAGCT   1755
ATGTGGTAGCACCTTGTCATGCTTGCGCGGCTCCCGGCGGTGAGGTGGCCGCCGTACCTGCTCGA
mCHERRY

GTACAAGTCCGGACTCAGATCTCGAGCTGATCCAAAAAAGAAGAGAAAGGTAGATCCAAAAAAGA   1820
CATGTTCAGGCCTGAGTCTAGAGCTCGACTAGGTTTTTTCTTCTCTTTCCATCTAGGTTTTTTCT
mCHERRY
                         130
Pro Lys Lys Lys Arg Lys Val Asp Pro Lys Lys
SV40 NLS                        SV40 NLS
```

```
AGAGAAAGGTAGATCCAAAAAAGAAGAGAAAGGTAGGATCCACCGGATCTAGATAAGAATTCTGC    1885
TCTCTTTCCATCTAGGTTTTTTCTTCTCTTTCCATCCTAGGTGGCCTAGATCTATTCTTAAGACG
```

mCHERRY

130 Lys Arg Lys Val Asp Pro Lys Lys Lys 130 Arg Lys Val Gly Ser Thr Gly Ser Arg *

SV40 NLS — SV40 NLS — (in frame with SV40 NLS) — β-globin poly(A) signal

```
AGTCGAGTCATCGCGATTAATTCACTCCTCAGGTGCAGGCTGCCTATCAGAAGGTGGTGGCTGGT    1950
TCAGCTCAGTAGCGCTAATTAAGTGAGGAGTCCACGTCCGACGGATAGTCTTCCACCACCGACCA
```

mCHERRY

β-globin poly(A) signal

```
GTGGCCAATGCCCTGGCTCACAAATACCACTGAGATCTTTTTCCCTCTGCCAAAAATTATGGGGA    2015
CACCGGTTACGGGACCGAGTGTTTATGGTGACTCTAGAAAAAGGGAGACGGTTTTTAATACCCCT
```

mCHERRY

β-globin poly(A) signal

```
CATCATGAAGCCCCTTGAGCATCTGACTTCTGGCTAATAAAGGAAATTTATTTTCATTGCAATAG    2080
GTAGTACTTCGGGGAACTCGTAGACTGAAGACCGATTATTTCCTTTAAATAAAAGTAACGTTATC
```

mCHERRY

β-globin poly(A) signal

```
TGTGTTGGAATTTTTTGTGTCTCTCACTCTCTGGATCTAACTAGTGAAGTTCCTATTCTCTAGAA    2145
ACACAACCTTAAAAAACACAGAGAGTGAGAGACCTAGATTGATCACTTCAAGGATAAGAGATCTT
```

mCHERRY — FRT

β-globin poly(A) signal

```
AGTATAGGAACTTCGAAGAGTACGAGGAGGaatttaagaggtgtattctgaggccacattgcttt    2210
TCATATCCTTGAAGCTTCTCATGCTCCTCCttaaattctccacataagactccggtgtaacgaaa
```

FRT — exon 6 poly(A)

```
gcatgccaataaataaatttttcttttagtgttgtgtagccaaaaattacaaatggaataaagttt    2275
cgtacggttatttatttaaaaagaaaatcacaacacatcggtttttaatgtttaccttatttcaaa
```

exon 6 poly(A)

```
tatcaaaatattgctaaaatatcagctttaaaatatgaaagtgctagattctgttattttcttct    2340
atagttttataacgattttatagtcgaaattttatactttcacgatctaagacaataaaagaaga
```

exon 6 poly(A)

```
tattttggatgaagtaccccaacctgtttacatttagcgataaaattatttttctatgatataat 2405
ataaaacctacttcatggggttggacaaatgtaaatcgctattttaataaaaagatactatatta
exon 6 poly(A)

ttgtaaatgtaaattattccgatctgacatatctgcattataataataggagaatagaagaactg 2470
aacatttacatttaataaggctagactgtatagacgtaatattattatcctcttatcttcttgac
exon 6 poly(A)

gtagccacagtggtgaaattggaaagagaactttcttcctgaaacctttgtcttaaaaatactca 2535
catcggtgtcaccactttaacctttctcttgaaagaaggactttggaaacagaatttttatgagt
exon 6 poly(A)

gctttcaatgtatcaaagatacaattaaataaaattttcaagcttctttaccattgtctgatttc 2600
cgaaagttacatagtttctatgttaatttattttaaaagttcgaagaaatggtaacagactaaag
exon 6 poly(A)   3' genomic region

tctttacgtcccccttttgtcatgccccactccttccaagcaccccctgtttttctaagctgcagtcc 2665
agaaatgcagggggaaacagtacggggtgaggaaggttcgtggggacaaaagattcgacgtcagg
3' genomic region

acaagtcaaatttggtgtataggcatgacttcatgcttcagatggctgcacagaacttttaacat 2730
tgttcagtttaaaccacatatccgtactgaagtacgaagtctaccgacgtgtcttgaaaattgta
3' genomic region

tttgaaccaacaatgaaaaatcagggtctttcatagaaaactattgatttgtagtgtctcctgaa 2795
aaacttggttgttactttttagtcccagaaagtatcttttgataactaaacatcacagaggactt
3' genomic region

aaagtaggtcatgaggcaatattagacccaaaaggcaagtttattctgtagctaagtaacagctg 2860
tttcatccagtactccgttataatctgggttttccgttcaaataagacatcgattcattgtcgac
3' genomic region

cctcttaccaaaggatcaggtgctccccagcttgtgtagggccccagcacatgcccaggcgtgct 2925
ggagaatggtttcctagtccacgaggggtcgaacacatcccggggtcgtgtacgggtccgcacga
3' genomic region

cacacaccccacctacataatggggacatcaaggaatttacttctggcccactttacttgcatac 2990
gtgtgtggggtggatgtattacccctgtagttccttaaatgaagaccgggtgaaatgaacgtatg
3' genomic region
```

```
tgcacctgcaggcatttgagtttgcccatcatgccttgtagccgttctccttgtcctgcaatggc    3055
acgtggacgtccgtaaactcaaacgggtagtacggaacatcggcaagaggaacaggacgttaccg
                          3' genomic region

caataccttgggacagtctgatagtgattgccaagcctgagactttctttgctggatttagcat    3120
gttatggaaccctgtcagactatcactaacggttcggactctgaaagaaacgacctaaatcgta
                          3' genomic region

tgcca   3'
             3125
acggt   5'
3' genomic region
```

5′ TTATCGGATCTTCGCTACCTTAGGACCGTTATAGTTAGCGGCCGCgccatggccctgtggatgcg 65
3′ AATAGCCTAGAAGCGATGGAATCCTGGCAATATCAATCGCCGGCGcggtaccgggacacctacgc
intron 1 exon 2

cctcctgcccctgctggcgctgctggccctctggggacctgacccagccgcagcctttgtgaacc 130
ggaggacggggacgaccgcgacgaccgggagacccctggactgggtcggcgtcggaaacacttgg
exon 2

aacacctgtgcggctcacacctggtggaagctctctacctagtgtgcggggaacgaggcttcttc 195
ttgtggacacgccgagtgtggaccaccttcgagagatggatcacacgccccttgctccgaagaag
exon 2

tacacacccaagacccgccgggaggcagaggacctgcagggtgagccaactgcccattgctgccc 260
atgtgtgggttctgggcggccctccgtctcctggacgtcccactcggttgacgggtaacgacggg
exon 2 intron 2

ctggccgcccccagccacccccctgctcctggcgctcccacccagcatgggcagaagggggcagga 325
gaccggcgggggtcggtgggggacgaggaccgcgagggtgggtcgtacccgtcttcccccgtcct
intron 2

ggctgccacccagcagggggtcaggtgcacttttttaaaaagaagttctcttggtcacgtcctaa 390
ccgacggtgggtcgtcccccagtccacgtgaaaaaatttttcttcaagagaaccagtgcaggatt
intron 2

aagtgaccagctccctgtggcccagtcagaatctcagcctgaggacggtgttggcttcggcagcc 455
ttcactggtcgagggacaccgggtcagtcttagagtcggactcctgccacaaccgaagccgtcgg
intron 2

ccgagatacatcagagggtgggcacgctcctccctccactcgcccctcaaacaaatgccccgcag 520
ggctctatgtagtctcccacccgtgcgaggagggaggtgagcggggagtttgtttacggggcgtc
intron 2

cccatttctccaccctcatttgatgaccgcagattcaagtgttttgttaagtaaagtcctgggtg 585
gggtaaagaggtgggagtaaactactggcgtctaagttcacaaaacaattcatttcaggacccac
intron 2

acctggggtcacagggtgccccacgctgcctgcctctgggcgaacaccccatcacgcccggagga 650
tggaccccagtgtcccacggggtgcgacggacggagacccgcttgtggggtagtgcgggcctcct
intron 2

gggcgtggctgcctgcctgagtgggccagacccctgtcgccaggcctcacggcagctccatagtc 715
cccgcaccgacggacggactcacccggtctggggacagcggtccggagtgccgtcgaggtatcag
intron 2

aggagatggggaagatgctggggacaggccctggggagaagtactgggatcacctgttcaggctc 780
tcctctaccccttctacgacccctgtccgggacccctcttcatgaccctagtggacaagtccgag
intron 2

ccactgtgacgctgccccggggcgggggaaggaggtgggacatgtgggcgttggggcctgtaggt 845
ggtgacactgcgacggggccccgcccccttcctccaccctgtacacccgcaaccccggacatcca
intron 2

ccacacccagtgtgggtgaccctccctctaacctgggtccagcccggctggagatgggtgggagt 910
ggtgtgggtcacacccactgggagggagattggacccaggtcgggccgacctctacccaccctca
intron 2

gcgacctagggctggcgggcaggcgggcactgtgtctccctgactgtgtcctcctgtgtccctct 975
cgctggatcccgaccgcccgtccgcccgtgacacagagggactgacacaggaggacacagggaga
intron 2

gcctcgccgctgttccggaacctgctctgcgcggcacgtcctggcagtggggcaggtggagctgg 1040
cggagcggcgacaaggccttggacgagacgcgccgtgcaggaccgtcaccccgtccacctcgacc
intron 2 exon 3

gcgggggccctggtgcaggcagcctgcagcccttggccctggaggggtccctgcagaagcgtggc 1105
cgcccccgggaccacgtccgtcggacgtcgggaaccgggacctccccagggacgtcttcgcaccg
exon 3

attgtggaacaatgctgtacAagcatctgctccctctaccagctggagaactactgcaacGGCAG 1170
taacaccttgttacgacatgTtcgtagacgagggagatggtcgacctcttgatgacgttgCCGTC
exon 3

TGGAGAGGGCAGAGGAAGTCTGCTAACATGCGGTGACGTCGAGGAGAATCCTGGCCCAGATCTCG 1235
ACCTCTCCCGTCTCCTTCAGACGATTGTACGCCACTGCAGCTCCTCTTAGGACCGGGTCTAGAGC
140 145 150 155
Glu Gly Arg Gly Ser Leu Leu Thr Cys Gly Asp Val Glu Glu Asn Pro Gly Pro Asp Leu
T2A

```
CCACCATGGTCGACTCATCACGTCGTAAGTGGAATAAGACAGGTCACGCAGTCAGAGCTATAGGT   1300
GGTGGTACCAGCTGAGTAGTGCAGCATTCACCTTATTCTGTCCAGTGCGTCAGTCTCGATATCCA
        1                 5                 10                15                20
Ala Thr Met Val Asp Ser Ser Arg Arg Lys Trp Asn Lys Thr Gly His Ala Val Arg Ala Ile Gly
GCaMP6
GCAMP6

CGGCTGAGCTCACTCGAGAACGTCTATATCAAGGCCGACAAGCAGAAGAACGGCATCAAGGCGAA   1365
GCCGACTCGAGTGAGCTCTTGCAGATATAGTTCCGGCTGTTCGTCTTCTTGCCGTAGTTCCGCTT
                25                30                35                40
Arg Leu Ser Ser Leu Glu Asn Val Tyr Ile Lys Ala Asp Lys Gln Lys Asn Gly Ile Lys Ala Asn
GCaMP6
GCAMP6

CTTCCACATCCGCCACAACATCGAGGACGGCGGCGTGCAGCTCGCCTACCACTACCAGCAGAACA   1430
GAAGGTGTAGGCGGTGTTGTAGCTCCTGCCGCCGCACGTCGAGCGGATGGTGATGGTCGTCTTGT
        45                50                55                60
Phe His Ile Arg His Asn Ile Glu Asp Gly Gly Val Gln Leu Ala Tyr His Tyr Gln Gln Asn
GCaMP6
GCAMP6

CCCCCATCGGCGACGGCCCCGTGCTGCTGCCCGACAACCACTACCTGAGCGTGCAGTCCAAACTT   1495
GGGGGTAGCCGCTGCCGGGGCACGACGACGGGCTGTTGGTGATGGACTCGCACGTCAGGTTTGAA
    65                70                75                80                85
Thr Pro Ile Gly Asp Gly Pro Val Leu Leu Pro Asp Asn His Tyr Leu Ser Val Gln Ser Lys Leu
GCaMP6
GCAMP6

TCGAAAGACCCCAACGAGAAGCGCGATCACATGGTCCTGCTGGAGTTCGTGACCGCCGCCGGGAT   1560
AGCTTTCTGGGGTTGCTCTTCGCGCTAGTGTACCAGGACGACCTCAAGCACTGGCGGCGGCCCTA
                90                95                100               105
Ser Lys Asp Pro Asn Glu Lys Arg Asp His Met Val Leu Leu Glu Phe Val Thr Ala Ala Gly Ile
GCaMP6
GCAMP6

CACTCTCGGCATGGACGAGCTGTACAAGGGCGGTACCGGAGGGAGCATGGTGAGCAAGGGCGAGG   1625
GTGAGAGCCGTACCTGCTCGACATGTTCCCGCCATGGCCTCCCTCGTACCACTCGTTCCCGCTCC
        110               115               120               125
Thr Leu Gly Met Asp Glu Leu Tyr Lys Gly Gly Thr Gly Gly Ser Met Val Ser Lys Gly Glu
GCaMP6
GCAMP6

AGCTGTTCACCGGGGTGGTGCCCATCCTGGTCGAGCTGGACGGCGACGTAAACGGCCACAAGTTC   1690
TCGACAAGTGGCCCCACCACGGGTAGGACCAGCTCGACCTGCCGCTGCATTTGCCGGTGTTCAAG
    130               135               140               145               150
Glu Leu Phe Thr Gly Val Val Pro Ile Leu Val Glu Leu Asp Gly Asp Val Asn Gly His Lys Phe
GCaMP6
GCAMP6
```

AGCGTGTCCGGCGAGGGTGAGGGCGATGCCACCTACGGCAAGCTGACCCTGAAGTTCATCTGCAC 1755
TCGCACAGGCCGCTCCCACTCCCGCTACGGTGGATGCCGTTCGACTGGGACTTCAAGTAGACGTG
155 160 165 170
Ser Val Ser Gly Glu Gly Glu Gly Asp Ala Thr Tyr Gly Lys Leu Thr Leu Lys Phe Ile Cys Thr
GCaMP6
GCAMP6

CACCGGCAAGCTGCCCGTGCCCTGGCCCACCCTCGTGACCACCCTGACCTACGGCGTGCAGTGCT 1820
GTGGCCGTTCGACGGGCACGGGACCGGGTGGGAGCACTGGTGGGACTGGATGCCGCACGTCACGA
175 180 185 190
Thr Gly Lys Leu Pro Val Pro Trp Pro Thr Leu Val Thr Thr Leu Thr Tyr Gly Val Gln Cys
GCaMP6
GCAMP6

TCAGCCGCTACCCCGACCACATGAAGCAGCACGACTTCTTCAAGTCCGCCATGCCCGAAGGCTAC 1885
AGTCGGCGATGGGGCTGGTGTACTTCGTCGTGCTGAAGAAGTTCAGGCGGTACGGGCTTCCGATG
195 200 205 210 215
Phe Ser Arg Tyr Pro Asp His Met Lys Gln His Asp Phe Phe Lys Ser Ala Met Pro Glu Gly Tyr
GCaMP6
GCAMP6

ATCCAGGAGCGCACCATCTTCTTCAAGGACGACGGCAACTACAAGACCCGCGCCGAGGTGAAGTT 1950
TAGGTCCTCGCGTGGTAGAAGAAGTTCCTGCTGCCGTTGATGTTCTGGGCGCGGCTCCACTTCAA
220 225 230 235
Ile Gln Glu Arg Thr Ile Phe Phe Lys Asp Asp Gly Asn Tyr Lys Thr Arg Ala Glu Val Lys Phe
GCaMP6
GCAMP6

CGAGGGCGACACCCTGGTGAACCGCATCGAGCTGAAGGGCATCGACTTCAAGGAGGACGGCAACA 2015
GCTCCCGCTGTGGGACCACTTGGCGTAGCTCGACTTCCCGTAGCTGAAGTTCCTCCTGCCGTTGT
240 245 250 255
Glu Gly Asp Thr Leu Val Asn Arg Ile Glu Leu Lys Gly Ile Asp Phe Lys Glu Asp Gly Asn
GCaMP6
GCAMP6

TCCTGGGGCACAAGCTGGAGTACAACCTGCCGGACCAACTGACTGAAGAGCAGATCGCAGAATTT 2080
AGGACCCCGTGTTCGACCTCATGTTGGACGGCCTGGTTGACTGACTTCTCGTCTAGCGTCTTAAA
260 265 270 275 280
Ile Leu Gly His Lys Leu Glu Tyr Asn Leu Pro Asp Gln Leu Thr Glu Glu Gln Ile Ala Glu Phe
GCaMP6
GCAMP6

AAAGAGGCTTTCTCCCTATTTGACAAGGACGGGGATGGGACAATAACAACCAAGGAGCTGGGGAC 2145
TTTCTCCGAAAGAGGGATAAACTGTTCCTGCCCCTACCCTGTTATTGTTGGTTCCTCGACCCCTG
285 290 295 300
Lys Glu Ala Phe Ser Leu Phe Asp Lys Asp Gly Asp Gly Thr Ile Thr Thr Lys Glu Leu Gly Thr
GCaMP6
GCAMP6

GGTGATGCGGTCTCTGGGGCAGAACCCCACAGAAGCAGAGCTGCAGGACATGATCAATGAAGTAG 2210

CCACTACGCCAGAGACCCCGTCTTGGGGTGTCTTCGTCTCGACGTCCTGTACTAGTTACTTCATC

305 310 315 320

Val Met Arg Ser Leu Gly Gln Asn Pro Thr Glu Ala Glu Leu Gln Asp Met Ile Asn Glu Val

GCaMP6

GCAMP6

ATGCCGACGGTGACGGCACAATCGACTTCCCTGAGTTCCTGACAATGATGGCAAGAAAAATGAAA 2275

TACGGCTGCCACTGCCGTGTTAGCTGAAGGGACTCAAGGACTGTTACTACCGTTCTTTTTACTTT

325 330 335 340 345

Asp Ala Asp Gly Asp Gly Thr Ile Asp Phe Pro Glu Phe Leu Thr Met Met Ala Arg Lys Met Lys

GCaMP6

GCAMP6

TACAGGGACACGGAAGAAGAAATTAGAGAAGCGTTCGGTGTGTTTGATAAGGATGGCAATGGCTA 2340

ATGTCCCTGTGCCTTCTTCTTTAATCTCTTCGCAAGCCACACAAACTATTCCTACCGTTACCGAT

350 355 360 365

Tyr Arg Asp Thr Glu Glu Glu Ile Arg Glu Ala Phe Gly Val Phe Asp Lys Asp Gly Asn Gly Tyr

GCaMP6

GCAMP6

CATCAGTGCAGCAGAGCTTCGCCACGTGATGACAAACCTTGGAGAGAAGTTAACAGATGAAGAGG 2405

GTAGTCACGTCGTCTCGAAGCGGTGCACTACTGTTTGGAACCTCTCTTCAATTGTCTACTTCTCC

370 375 380 385

Ile Ser Ala Ala Glu Leu Arg His Val Met Thr Asn Leu Gly Glu Lys Leu Thr Asp Glu Glu

GCaMP6

GCAMP6

TTGATGAAATGATCAGGGAAGCAGACATCGATGGGGATGGTCAGGTAAACTACGAAGAGTTTGTA 2470

AACTACTTTACTAGTCCCTTCGTCTGTAGCTACCCCTACCAGTCCATTTGATGCTTCTCAAACAT

390 395 400 405 410

Val Asp Glu Met Ile Arg Glu Ala Asp Ile Asp Gly Asp Gly Gln Val Asn Tyr Glu Glu Phe Val

GCaMP6

GCAMP6

CAAATGATGACAGCGAAGTGAGCGGCCGCGACTCTAGATCATAATCAGCCATACCACATTTGTAG 2535

GTTTACTACTGTCGCTTCACTCGCCGGCGCTGAGATCTAGTATTAGTCGGTATGGTGTAAACATC

415 420 425 430

Gln Met Met Thr Ala Lys * Ala Ala Ala Thr Leu Asp His Asn Gln Pro Tyr His Ile Cys Arg

GCaMP6

GCAMP6

SV40 poly(A)

AGGTTTTACTTGCTTTAAAAAACCTCCCACACCTCCCCCTGAACCTGAAACATAAAATGAATGCA 2600

TCCAAAATGAACGAAATTTTTTGGAGGGTGTGGAGGGGGACTTGGACTTTGTATTTTACTTACGT

435 440 445 450

Gly Phe Thr Cys Phe Lys Lys Pro Pro Thr Pro Pro Pro Glu Pro Glu Thr * Asn Glu Cys

GCAMP6

SV40 poly(A)

```
ATTGTTGTTGTTAACTTGTTTATTGCAGCTTATAATGGTTACAAATAAAGCAATAGCATCACAAA   2665
TAACAACAACAATTGAACAAATAACGTCGAATATTACCAATGTTTATTTCGTTATCGTAGTGTTT
 455                 460                 465                 470                 475
Asn Cys Cys Cys * Leu Val Tyr Cys Ser Leu * Trp Leu Gln Ile Lys Gln * His His Lys
GCAMP6
SV40 poly(A)

TTTCACAAATAAAGCATTTTTTTCACTGCATTCTAGTTGTGGTTTGTCCAAACTCATCAATGTAT   2730
AAAGTGTTTATTTCGTAAAAAAAGTGACGTAAGATCAACACCAAACAGGTTTGAGTAGTTACATA
             480                 485                 490                 495
Phe His Lys * Ser Ile Phe Phe Thr Ala Phe * Leu Trp Phe Val Gln Thr His Gln Cys Ile
GCAMP6
SV40 poly(A)

CTTAAGGCTAGAGTCAACGAAGTTCCTATACTTTCTAGAGAATAGGAACTTCGGAATAGGAACTT   2795
GAATTCCGATCTCAGTTGCTTCAAGGATATGAAAGATCTCTTATCCTTGAAGCCTTATCCTTGAA
Leu Arg Leu Glu Ser Thr Lys Phe Leu Tyr Phe Leu Glu Asn Arg Asn Phe Gly Ile Gly Thr
(in frame with GCAMP6)
GCAMP6
FRT
SV40 poly(A)

CGTTGACCTAGAGTCGacgcagcccgcagAcagccccacacccgccgcctcctgcaccgagagag   2860
GCAACTGGATCTCAGCtgcgtcgggcgtcTgtcggggtgtgggcggcggaggacgtggctctctc
Ser Leu Thr *
exon 3 3' UTR
FRT

atggaataaagcccttgaaccagccctgctgtgccgtctgtgtgtcttgggggccctgggccaag   2925
taccttatttcgggaacttggtcgggacgacacggcagacacacagaacccccgggacccggttc
exon 3 3' UTR
3' genomic region

ccccacttcccggcactgttgtgagcccctcccagctctctccacgctctctgggtgcccacagg   2990
ggggtgaagggccgtgacaacactcggggagggtcgagagaggtgcgagagacccacgggtgtcc
3' genomic region

tgccaacgccggccaggcccagcatgcagtggctctccccaaagcggccatgcctgtcggctgcc   3055
acggttgcggccggtccgggtcgtacgtcaccgagaggggtttcgccggtacggacagccgacgg
3' genomic region

tgctgcccccaccctgtggctcagggtccagtatgggagctgcgggggtctctgaggggccaggg   3120
acgacgggggtgggacaccgagtcccaggtcataccctcgacgcccccagagactccccggtccc
3' genomic region
```

gtggtggggccactgagaagtgacttcttgttcagtagctctggactcttggagtccccagagac 3185
caccaccccggtgactcttcactgaagaacaagtcatcgagacctgagaacctcaggggtctctg
3' genomic region

cttgttcaggaaagggaatgagaacattccagcaatttcccccacctagccctcccaggttct 3250
gaacaagtcctttcccttactcttgtaaggtcgttaaagggggtggatcgggagggtccaaga
3' genomic region

attttagagttatttctgatggagtccctgtggagggaggaggctgggctgagggaggggtcc 3315
taaaaatctcaataaagactacctcagggacacctccctcctccgacccgactccctccccagg
3' genomic region

tgcagggcgggggctgggaaggtggggagaggctgccgagagccacccgctatccccagctctg 3380
acgtcccgcccccgacccttccacccctctccgacggctctcggtgggcgataggggtcgagac
3' genomic region

ggcagccccgggacagtcacacaccctggcctcgcggcccaagctggcagccgtctgcagccaca 3445
ccgtcggggccctgtcagtgtgtgggaccggagcgccgggttcgaccgtcggcagacgtcggtgt
3' genomic region

gcttatgccagcccaggtccagccagacacctgagggacccactggtgccttggaggaagcagga 3510
cgaatacggtcgggtccaggtcggtctgtggactccctgggtgaccacggaacctccttcgtcct
3' genomic region

gaggtcagatggcaccatgagctggggcaggtgcagggaccgtggcagcacctggcagggcctca 3575
ctccagtctaccgtggtactcgaccccgtccacgtccctggcaccgtcgtggaccgtcccggagt
3' genomic region

gaacccatgccttgggcaccccggccatgaggccctgaggattgcagcccaggagaagcagggaa 3640
cttgggtacggaacccgtggggccggtactccgggactcctaacgtcgggtcctcttcgtccctt
3' genomic region

ccgccagggccacaggggcagagaccagggccagggtcccctgcagccccttagcccacccct 3705
ggcggtcccggtgtccccgtctctggtcccggtcccaggggacgtcggggaatcgggtgggga
3' genomic region

cccagtaagcagggctgcttggctggcttcctttgctacagacctgctgctcacccagaagggcc 3770
gggtcattcgtcccgacgaaccgaccgaaggaaacgatgtctggacgacgagtgggtcttcccgg
3' genomic region

```
cacgggccctggtgacaaggtcgttgtggctccaggtccttggggtcctgacacagagcctctt
                                                                      3835
gtgcccgggaccactgttccagcaacaccgaggtccaggaaccccaggactgtgtctcggagaa
                       3' genomic region

ctgcagcacccctgaggacagggtggctccgctgggcacccagcctagtgggcagacgagaacct
                                                                      3900
gacgtcgtggggactcctgtcccaccgaggcgacccgtgggtcggatcacccgtctgctcttgga
                       3' genomic region

aggggctgcctgggcctactgtggcctgggaggtcagcgggtgaccctagctaccctgtggctgg
                                                                      3965
tccccgacggacccggatgacaccggaccctccagtcgcccactgggatcgatgggacaccgacc
                       3' genomic region

gccagtctgcctgccacccaggccaaaccaatGCGGCCGCCATGGTCATAGCTGTTTGACGTCAG
                                                                      4030
cggtcagacggacggtgggtccggtttggttaCGCCGGCGGTACCAGTATCGACAAACTGCAGTC
                       3' genomic region

GTGGCACTTTTCG    3′
                   4043
CACCGTGAAAAGC-(P) 5′
3' genomic region
```

Sequence: SUP FILE 4 MAFA mCHERRY REPORTER copy.dna (Circular / 3457 bp)
Features: 11 total

5′ gcgcggccgggcgcgggcccgggcgatggccgcggagctggcgatgggcgccgagctgcccagc 65
3′ cgcgccggcccgcgcccgggcccgctaccggcgcctcgaccgctacccgcggctcgacgggtcg

1 Met Ala Ala Glu 5 Leu Ala Met Gly Ala 10 Glu Leu Pro Ser

exon 1 5' UTR | exon 1 CDS

agcccgctggccatcgagtacgtcaacgacttcgacctgatgaagttcgaggtgaagaaggagcc 130
tcgggcgaccggtagctcatgcagttgctgaagctggactacttcaagctccacttcttcctcgg

Ser 15 Pro Leu Ala Ile Glu 20 Tyr Val Asn Asp Phe 25 Asp Leu Met Lys Phe 30 Glu Val Lys Lys Glu 35 Pro

exon 1 CDS

tcccgaggccgagcgcttctgccaccgcctgccgccaggctcgctgtcctcgacgccgctcagca 195
agggctccggctcgcgaagacggtggcggacggcggtccgagcgacaggagctgcggcgagtcgt

Pro Glu Ala Glu 40 Arg Phe Cys His Arg 45 Leu Pro Pro Gly Ser 50 Leu Ser Ser Thr Pro 55 Leu Ser

exon 1 CDS

cgccctgctcctccgtgccctcctcgcccagcttctgcgcgcccagcccgggcaccggcggcggc 260
gcgggacgaggaggcacgggaggagcgggtcgaagacgcgcgggtcgggcccgtggccgccgccg

Thr Pro Cys 60 Ser Ser Val Pro Ser 65 Ser Pro Ser Phe Cys 70 Ala Pro Ser Pro Gly 75 Thr Gly Gly Gly

exon 1 CDS

ggcggcgcgggggcggcggcggctcgtctcaggccgggggcgcccccgggccgccgagcggggg 325
ccgccgcgcccccgccgccgccgagcagagtccggcccccgcgggggcccggcggctcgccccc

Gly 80 Gly Ala Gly Gly Gly 85 Gly Gly Ser Ser Gln 90 Ala Gly Gly Ala Pro 95 Gly Pro Pro Ser Gly 100 Gly

exon 1 CDS

ccccggcgccgtcgggggcacctcggggaagccggcgctggaggatctgtactggatgagcggct 390
ggggccgcggcagcccccgtggagccccttcggccgcgacctcctagacatgacctactcgccga

Pro Gly Ala Val 105 Gly Gly Thr Ser Gly 110 Lys Pro Ala Leu Glu 115 Asp Leu Tyr Trp Met 120 Ser Gly

exon 1 CDS

accagcatcacctcaaccccgaggcgctcaacctgacgcccgaggacgcggtggaggcgctcatc 455
tggtcgtagtggagttggggctccgcgagttggactgcgggctcctgcgccacctccgcgagtag

Tyr Gln His 125 His Leu Asn Pro Glu 130 Ala Leu Asn Leu Thr 135 Pro Glu Asp Ala Val 140 Glu Ala Leu Ile

exon 1 CDS

ggcagcggccaccacggcgcgcaccacggcgcgcaccacccggcggccgccgcagcctacgaggc 520
ccgtcgccggtggtgccgcgcgtggtgccgcgcgtggtgggccgccggcggcgtcggatgctccg

Gly 145 Ser Gly His His Gly 150 Ala His His Gly Ala 155 His His Pro Ala Ala 160 Ala Ala Ala Tyr Glu 165 Ala

exon 1 CDS

```
tttccgcggcccgggcttcgcgggcggcggcggagcggacgacatgggcgccggccaccaccacg   585
aaaggcgccgggcccgaagcgcccgccgccgcctcgcctgctgtacccgcggccggtggtggtgc
          170                175                180                185
 Phe  Arg  Gly  Pro  Gly  Phe  Ala  Gly  Gly  Gly  Gly  Ala  Asp  Asp  Met  Gly  Ala  Gly  His  His  His
exon 1 CDS >
```

```
gcgcgcaccacgccgcccaccaCcaccacgccgcccaccaccaccaccaccaccaccaccatggc   650
cgcgcgtggtgcggcgggtggtGgtggtgcggcgggtggtggtggtggtggtggtggtggtaccg
          190                195                200                205
Gly  Ala  His  His  Ala  Ala  His  His  His  His  Ala  Ala  His  His  His  His  His  His  His  His  His  Gly
exon 1 CDS >
```

```
ggcgcgggacacggcggtggcgcgggccaccacgtgcgcctggaggagcgcttctccgacgacca   715
ccgcgccctgtgccgccaccgcgcccggtggtgcacgcggacctcctcgcgaagaggctgctggt
     210                215                220                225                230
 Gly  Ala  Gly  His  Gly  Gly  Gly  Ala  Gly  His  His  Val  Arg  Leu  Glu  Glu  Arg  Phe  Ser  Asp  Asp  Gln
exon 1 CDS >
```

```
gctggtgtccatgtcggtgcgcgagctgaaccggcagctccgcggcttcagcaaggaggaggtca   780
cgaccacaggtacagccacgcgctcgacttggccgtcgaggcgccgaagtcgttcctcctccagt
          235                240                245                250
 Leu  Val  Ser  Met  Ser  Val  Arg  Glu  Leu  Asn  Arg  Gln  Leu  Arg  Gly  Phe  Ser  Lys  Glu  Glu  Val
exon 1 CDS >
```

```
tccggctcaagcagaagcggcgcacgctcaagaaccgcggctacgcgcagtcctgccgcttcaag   845
aggccgagttcgtcttcgccgcgtgcgagttcttggcgccgatgcgcgtcaggacggcgaagttc
          255                260                265                270
Ile  Arg  Leu  Lys  Gln  Lys  Arg  Arg  Thr  Leu  Lys  Asn  Arg  Gly  Tyr  Ala  Gln  Ser  Cys  Arg  Phe  Lys
exon 1 CDS >
```

```
cgggtgcagcagcggcacattctggagagcgagaagtgccaactccagagccaggtggagcagct   910
gcccacgtcgtcgccgtgtaagacctctcgctcttcacggttgaggtctcggtccacctcgtcga
     275                280                285                290                295
 Arg  Val  Gln  Gln  Arg  His  Ile  Leu  Glu  Ser  Glu  Lys  Cys  Gln  Leu  Gln  Ser  Gln  Val  Glu  Gln  Leu
exon 1 CDS >
```

```
gaagctggaggtggggcgcctggccaaagagcgggacctgtacaaggagaaatacgagaagctgg   975
cttcgacctccaccccgcggaccggtttctcgccctggacatgttcctctttatgctcttcgacc
          300                305                310                315
 Lys  Leu  Glu  Val  Gly  Arg  Leu  Ala  Lys  Glu  Arg  Asp  Leu  Tyr  Lys  Glu  Lys  Tyr  Glu  Lys  Leu
exon 1 CDS >
```

```
cgggccggggcggcccccgggagcgcgggcggggccggtttcccgcgggagccttcgccgccgcag   1040
gcccggccccgccgggggccctcgcgcccgccccggccaaagggcgccctcggaagcggcggcgtc
          320                325                330                335
Ala  Gly  Arg  Gly  Gly  Pro  Gly  Ser  Ala  Gly  Gly  Ala  Gly  Phe  Pro  Arg  Glu  Pro  Ser  Pro  Pro  Gln
exon 1 CDS >
```

gccggtcccggcggggccaagggcacggccgacttcttcctggagggcagaggaagtcttctaac 1105
cggccagggccgccccggttcccgtgccggctgaagaaggacctcccgtctccttcagaagattg

340 345 350 | 140 145
Ala Gly Pro Gly Gly Ala Lys Gly Thr Ala Asp Phe Phe Leu | Glu Gly Arg Gly Ser Leu Leu Thr
exon 1 CDS | T2A

atgcggtgacgtggaggagaatcccggccctATGGTGAGCAAGGGCGAGGAGGATAACATGGCCA 1170
tacgccactgcacctcctcttagggccgggaTACCACTCGTTCCCGCTCCTCCTATTGTACCGGT

150 155 | 1 5 10
Cys Gly Asp Val Glu Glu Asn Pro Gly Pro | Met Val Ser Lys Gly Glu Glu Asp Asn Met Ala
T2A | mCHERRY

TCATCAAGGAGTTCATGCGCTTCAAGGTGCACATGGAGGGCTCCGTGAACGGCCACGAGTTCGAG 1235
AGTAGTTCCTCAAGTACGCGAAGTTCCACGTGTACCTCCCGAGGCACTTGCCGGTGCTCAAGCTC

15 20 25 30
Ile Ile Lys Glu Phe Met Arg Phe Lys Val His Met Glu Gly Ser Val Asn Gly His Glu Phe Glu
mCHERRY

ATCGAGGGCGAGGGCGAGGGCCGCCCCTACGAGGGCACCCAGACCGCCAAGCTGAAGGTGACCAA 1300
TAGCTCCCGCTCCCGCTCCCGGCGGGGATGCTCCCGTGGGTCTGGCGGTTCGACTTCCACTGGTT

35 40 45 50 55
Ile Glu Gly Glu Gly Glu Gly Arg Pro Tyr Glu Gly Thr Gln Thr Ala Lys Leu Lys Val Thr Lys
mCHERRY

GGGTGGCCCCCTGCCCTTCGCCTGGGACATCCTGTCCCCTCAGTTCATGTACGGCTCCAAGGCCT 1365
CCCACCGGGGGACGGGAAGCGGACCCTGTAGGACAGGGGAGTCAAGTACATGCCGAGGTTCCGGA

60 65 70 75
Gly Gly Pro Leu Pro Phe Ala Trp Asp Ile Leu Ser Pro Gln Phe Met Tyr Gly Ser Lys Ala
mCHERRY

ACGTGAAGCACCCCGCCGACATCCCCGACTACTTGAAGCTGTCCTTCCCCGAGGGCTTCAAGTGG 1430
TGCACTTCGTGGGGCGGCTGTAGGGGCTGATGAACTTCGACAGGAAGGGGCTCCCGAAGTTCACC

80 85 90 95
Tyr Val Lys His Pro Ala Asp Ile Pro Asp Tyr Leu Lys Leu Ser Phe Pro Glu Gly Phe Lys Trp
mCHERRY

GAGCGCGTGATGAACTTCGAGGACGGCGGCGTGGTGACCGTGACCCAGGACTCCTCCCTGCAGGA 1495
CTCGCGCACTACTTGAAGCTCCTGCCGCCGCACCACTGGCACTGGGTCCTGAGGAGGGACGTCCT

100 105 110 115 120
Glu Arg Val Met Asn Phe Glu Asp Gly Gly Val Val Thr Val Thr Gln Asp Ser Ser Leu Gln Asp
mCHERRY

CGGCGAGTTCATCTACAAGGTGAAGCTGCGCGGCACCAACTTCCCCTCCGACGGCCCCGTAATGC 1560
GCCGCTCAAGTAGATGTTCCACTTCGACGCGCCGTGGTTGAAGGGGAGGCTGCCGGGGCATTACG

125 130 135 140
Gly Glu Phe Ile Tyr Lys Val Lys Leu Arg Gly Thr Asn Phe Pro Ser Asp Gly Pro Val Met
mCHERRY

AGAAGAAGACCATGGGCTGGGAGGCCTCCTCCGAGCGGATGTACCCCGAGGACGGCGCCCTGAAG 1625
TCTTCTTCTGGTACCCGACCCTCCGGAGGAGGCTCGCCTACATGGGGCTCCTGCCGCGGGACTTC
145 150 155 160
Gln Lys Lys Thr Met Gly Trp Glu Ala Ser Ser Glu Arg Met Tyr Pro Glu Asp Gly Ala Leu Lys
mCHERRY

GGCGAGATCAAGCAGAGGCTGAAGCTGAAGGACGGCGGCCACTACGACGCTGAGGTCAAGACCAC 1690
CCGCTCTAGTTCGTCTCCGACTTCGACTTCCTGCCGCCGGTGATGCTGCGACTCCAGTTCTGGTG
165 170 175 180 185
Gly Glu Ile Lys Gln Arg Leu Lys Leu Lys Asp Gly Gly His Tyr Asp Ala Glu Val Lys Thr Thr
mCHERRY

CTACAAGGCCAAGAAGCCCGTGCAGCTGCCCGGCGCCTACAACGTCAACATCAAGTTGGACATCA 1755
GATGTTCCGGTTCTTCGGGCACGTCGACGGGCCGCGGATGTTGCAGTTGTAGTTCAACCTGTAGT
190 195 200 205
Tyr Lys Ala Lys Lys Pro Val Gln Leu Pro Gly Ala Tyr Asn Val Asn Ile Lys Leu Asp Ile
mCHERRY

CCTCCCACAACGAGGACTACACCATCGTGGAACAGTACGAACGCGCCGAGGGCCGCCACTCCACC 1820
GGAGGGTGTTGCTCCTGATGTGGTAGCACCTTGTCATGCTTGCGCGGCTCCCGGCGGTGAGGTGG
210 215 220 225
Thr Ser His Asn Glu Asp Tyr Thr Ile Val Glu Gln Tyr Glu Arg Ala Glu Gly Arg His Ser Thr
mCHERRY

GGCGGCATGGACGAGCTGTACAAGTCCGGACTCAGATCTCGAGCTGATCCAAAAAAGAAGAGAAA 1885
CCGCCGTACCTGCTCGACATGTTCAGGCCTGAGTCTAGAGCTCGACTAGGTTTTTTCTTCTCTTT
230 235 240 245 250
Gly Gly Met Asp Glu Leu Tyr Lys Ser Gly Leu Arg Ser Arg Ala Asp Pro Lys Lys Lys Arg Lys
mCHERRY

130
Pro Lys Lys Lys Arg Lys
SV40 NLS

GGTAGATCCAAAAAAGAAGAGAAAGGTAGATCCAAAAAAGAAGAGAAAGGTAGGATCCACCGGAT 1950
CCATCTAGGTTTTTTCTTCTCTTTCCATCTAGGTTTTTTCTTCTCTTTCCATCCTAGGTGGCCTA
255 260 265 270
Val Asp Pro Lys Lys Lys Arg Lys Val Asp Pro Lys Lys Lys Arg Lys Val Gly Ser Thr Gly
mCHERRY

130 130
Val Asp Pro Lys Lys Lys Arg Lys Val Asp Pro Lys Lys Lys Arg Lys Val Gly Ser Thr Gly
SV40 NLS SV40 NLS
SV40 NLS

CTAGATAAGAATTCTGCAGTCGAGTCATCGCGATTAATTCACTCCTCAGGTGCAGGCTGCCTATC 2015
GATCTATTCTTAAGACGTCAGCTCAGTAGCGCTAATTAAGTGAGGAGTCCACGTCCGACGGATAG
275 280 285 290
Ser Arg * Glu Phe Cys Ser Arg Val Ile Ala Ile Asn Ser Leu Leu Arg Cys Arg Leu Pro Ile
mCHERRY

Ser Arg *
β-globin poly(A)

STOP

```
AGAAGGTGGTGGCTGGTGTGGCCAATGCCCTGGCTCACAAATACCACTGAGATCTTTTTCCCTCT   2080
TCTTCCACCACCGACCACACCGGTTACGGGACCGAGTGTTTATGGTGACTCTAGAAAAAGGGAGA
    295        300        305        310        315
Arg Arg Trp Trp Leu Val Trp Pro Met Pro Trp Leu Thr Asn Thr Thr Glu Ile Phe Phe Pro Leu
mCHERRY
β-globin poly(A)

GCCAAAAATTATGGGGACATCATGAAGCCCCTTGAGCATCTGACTTCTGGCTAATAAAGGAAATT   2145
CGGTTTTTAATACCCCTGTAGTACTTCGGGGAACTCGTAGACTGAAGACCGATTATTTCCTTTAA
    320        325        330        335
Pro Lys Ile Met Gly Thr Ser * Ser Pro Leu Ser Ile * Leu Leu Ala Asn Lys Gly Asn
mCHERRY
β-globin poly(A)

TATTTTCATTGCAATAGTGTGTTGGAATTTTTTGTGTCTCTCACTCTCTGGATCTAACTAGTGAA   2210
ATAAAAGTAACGTTATCACACAACCTTAAAAAACACAGAGAGTGAGAGACCTAGATTGATCACTT
    340        345        350
Leu Phe Ser Leu Gln * Cys Val Gly Ile Phe Cys Val Ser His Ser Leu Asp Leu Thr Ser Glu
mCHERRY
β-globin poly(A)
FRT

GTTCCTATTCTCTAGAAAGTATAGGAACTTCGAAGAGTACGAGGAGGgcgccggaccccgagccc   2275
CAAGGATAAGAGATCTTTCATATCCTTGAAGCTTCTCATGCTCCTCCcgcggcctggggctcggg
Val Pro Ile Leu *
exon 1 poly(A)
FRT

gcgccgccgtcgccggggacaagttcgcgcaggcctctcggggcctcggctcggactccgcggta   2340
cgcggcggcagcggcccctgttcaagcgcgtccggagagccccggagccgagcctgaggcgccat
exon 1 poly(A)

caggacgtggacaccaggcccggcccggccgtgctggccccggtgccaagtctgcgggcgcgggg   2405
gtcctgcacctgtggtccgggccgggccggcacgaccggggccacggttcagacgcccgcgcccc
exon 1 poly(A)

ctggaggccccttcgctcccggtccccgttcgcgcgcgtcggcccgggtcgccgtcctgaggttg   2470
gacctccggggaagcgagggccaggggcaagcgcgcgcagccgggcccagcggcaggactccaac
exon 1 poly(A)

agcggagaacggtgatttctaaggaaacttgagccaggtctaacttctttccaagcgtccgcttg   2535
tcgcctcttgccactaaagattcctttgaactcggtccagattgaagaaaggttcgcaggcgaac
exon 1 poly(A)
```

```
tacatacgttgaacgtggttctccgttcccaccttcgccctgccagcctagagggaccgcgctgc
                                                                    2600
atgtatgcaacttgcaccaagaggcaagggtggaagcgggacggtcggatctccctggcgcgacg
exon 1 poly(A)

cgtcccttcccgggtggcccctgcctgcccccgccctccttcgttctcttctcagcctccctttc
                                                                    2665
gcagggaagggcccaccggggacggacgggggcgggaggaagcaagagaagagtcggagggaaag
exon 1 poly(A)

cttgccttttttaacttcccctccccgttttaaaatcggtcttattttcgaagtatttataatta
                                                                    2730
gaacggaaaaaattgaaggggaggggcaaaattttagccagaataaaagcttcataaatattaat
exon 1 poly(A)

ttatgcttggtgattagaaaagaaaaccttggaggaagccccttctttccccagccggggtccgc
                                                                    2795
aatacgaaccactaatcttttcttttggaacctccttcggggaagaaaggggtcggccccaggcg
exon 1 poly(A)

cctcagtcgcgagtcacagcatgagtcgctcgccaggaggggcccggcccctgcctgccccctcc
                                                                    2860
ggagtcagcgctcagtgtcgtactcagcgagcggtcctccccgggccggggacggacgggggagg
exon 1 poly(A)

ccgcttgcccccgaccctgctaccggcgttccttggaggtcgaagccagggacgtcacccgtgct
                                                                    2925
ggcgaacgggggctgggacgatggccgcaaggaacctccagcttcggtccctgcagtgggcacga
exon 1 poly(A)

gtgtccaggcctgctgtcctactatgctcaaccgggggtggggggaggggggtgagtcctgtgct
                                                                    2990
cacaggtccggacgacaggatgatacgagttggcccccacccccctccccccactcaggacacga
exon 1 poly(A)

cagtcgggtgggggctggcccggatcccgagctgctgtctctctatgcaccagaacatatctgta
                                                                    3055
gtcagcccacccccgaccgggcctagggctcgacgacagagagatacgtggtcttgtatagacat
exon 1 poly(A)

actcctggggaaatacatcttgttttaaccttcaagagaagtgaaagaaaaaagtaatgcacagt
                                                                    3120
tgaggacccctttatgtagaacaaaattggaagttctcttcactttcttttttcattacgtgtca
exon 1 poly(A)

atttctagcagaaaattttttttttttaagaggaggcttgggccagagccttctggcatggggcgg
                                                                    3185
taaagatcgtcttttaaaaaaaaaaattctcctccgaacccggtctcggaagaccgtaccccgcc
exon 1 poly(A)

gtggagaaagtgtttttattttaatttaaattgtgtttcgttttgtttgtggaatctttctttaa
                                                                    3250
cacctctttcacaaaaataaaattaaatttaacacaaagcaaaacaaacaccttagaaagaaatt
exon 1 poly(A)
```

```
tgcttcgtcgctctttggactagccgggagagagggcgaggaggcgggtgctccaggccctgtag   3315
acgaagcagcgagaaacctgatcggccctctctcccgctcctccgcccacgaggtccgggacatc
exon 1 poly(A)

gctgggccaggcgcctggggggatctgcccgttttcggaggccctcaggggccatcagtgggattc   3380
cgacccggtccgcggacccccctagacgggcaaaagcctccgggagtccccggtagtcaccctaag
exon 1 poly(A)

cagccgctccacacccctcccctgagcactcggagtggaaggcgcgccgactcgttgaaagtttt   3445
gtcggcgaggtgtggggaggggactcgtgagcctcaccttccgcgcggctgagcaactttcaaaa
exon 1 poly(A)

gttgtgtagttg     3′
             ••• 3457
caacacatcaac     5′
exon 1 poly(A)
```

H1 *INS* locus (wild type)

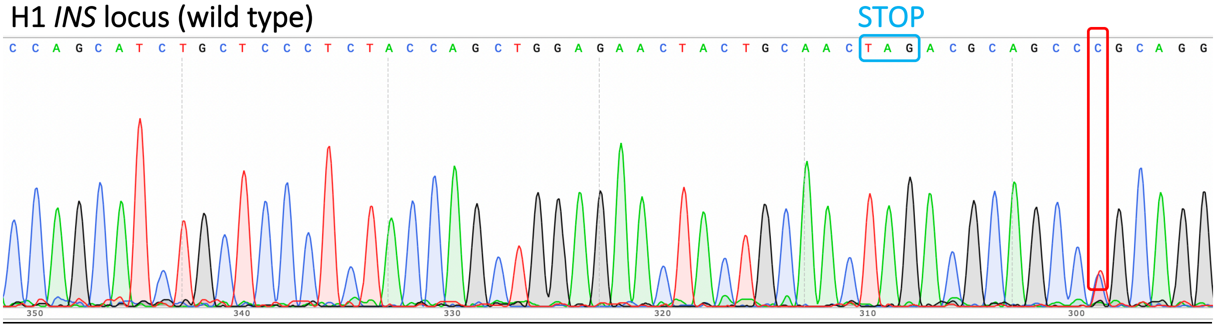

→ Parental H1 line carries a heterozygous C>T mutation after the STOP codon

wild type (untargeted) *INS* locus in *INS* *eGFP* clone # 36-1

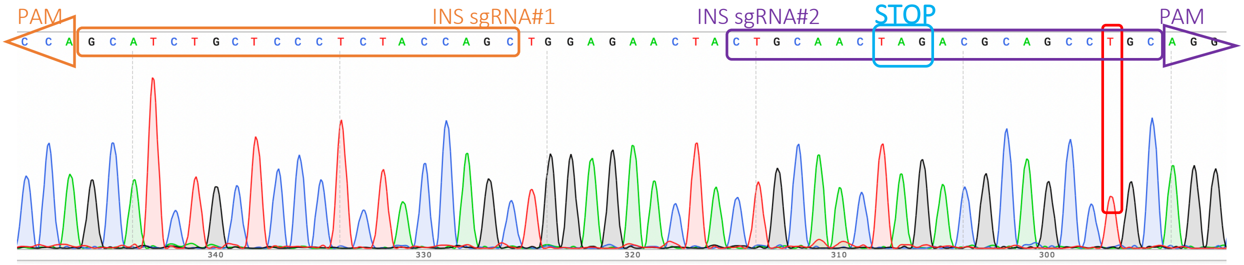

→ wild type *INS* locus is intact carrying the T SNP in the 3'UTR behind the STOP codon

wild type (untargeted) *INS* locus in *INS* *GCaMP6* clone # 96-2

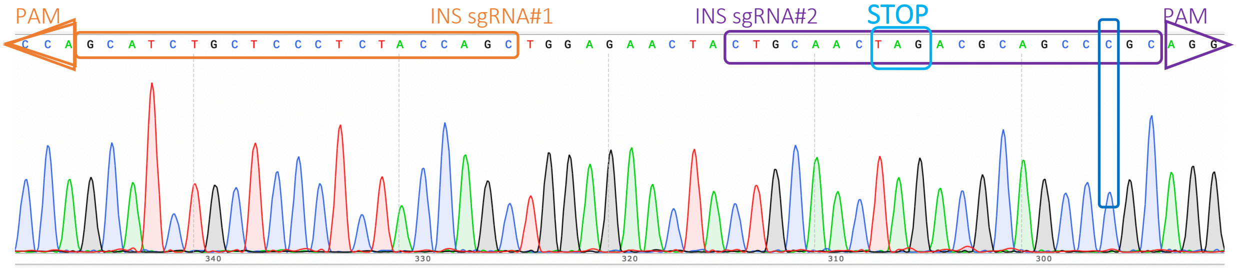

→ wild type *INS* locus is intact carrying the wild type SNP in the 3'UTR behind the STOP codon

MAFA locus in the *INS* ^{eGFP} clone # 36-1

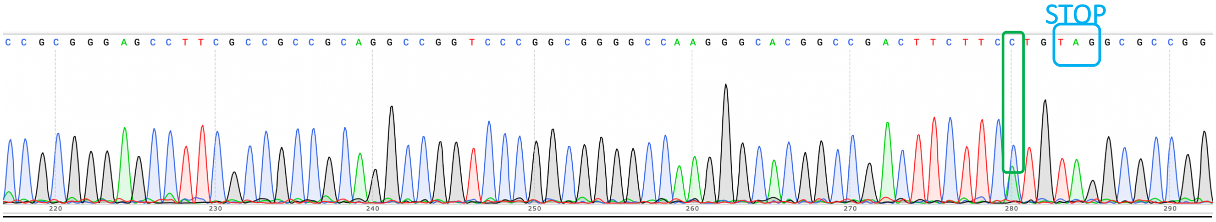

Parental line carries a heterozygous C>A mutation (L353M) in the codon before the STOP

Wild type (untargeted) *MAFA* locus in the *INS* ^{eGFP} / *MAFA* ^{mCherry} clone # 63-1

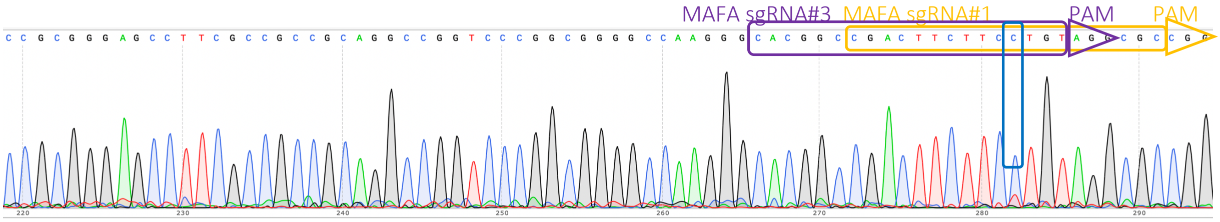

→ wild type *MAFA* locus is intact carrying the wild type SNP in the codon before the STOP

GCG locus in the *INS* ^{eGFP} clone # 36-1

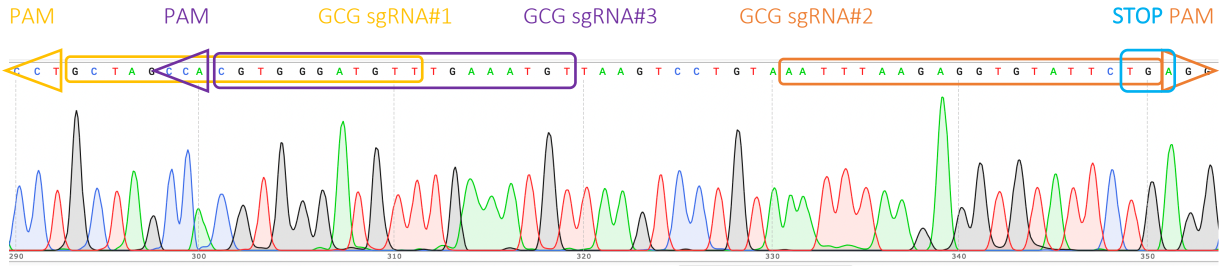

Wild type (untargeted) *GCG* locus in the *INS* ^{eGFP} / *GCG* ^{mCherry} clone # 24-8

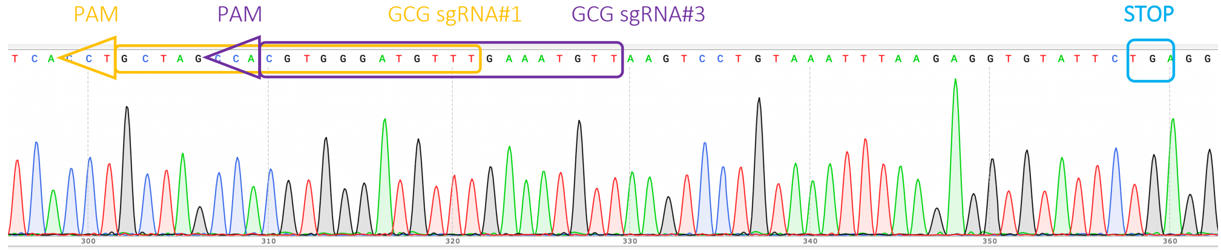

→ wild type *GCG* locus is intact
