# Supplementary material for: Generation and application of novel hES cell reporter lines for the differentiation and maturation of hPS cell-derived islet-like clusters
Source: Sci Rep. 2024 Aug 27;14:19863. doi: 10.1038/s41598-024-69645-4 (PMC11350089; doi:10.1038/s41598-024-69645-4)
Supplement: Supplementary file 3 — Supplementary Tables. [file 41598_2024_69645_MOESM3_ESM.pdf]

Allele	DNA fragments	Plasmid	Reference	Provider	Form
	pDTA-TK (plasmid backbone)	pDTA-TK	Nam and Benezra 2009	Addgene 22677.	NotI linearised
	5' INS HA	hINS 5pHA	this work	custome made, IDT	restriction fragment
INS ^{eGFP}	T2A-eGFP	SpCas9-2A-GFP	Ran et al. 2013	Addgene 48138.	PCR product
	PGK-Neomycin	PGKneoF2L2DTA	Hoch and Soriano 2006	Addgene 13445.	PCR product
	3' INS HA	hINS 3pHA	this work	custome made, IDT	restriction fragment
	pDTA-TK (plasmid backbone)	modified pDTA-TK*	this work	this work	NotI linearised
	5' GCG HA	genomic PCR fragment	this work	this work	PCR product
GCG ^{mCHERRY}	T2A-mCHERRY	pGEMT_EGFPbait-T2A-Cherry-3xNLS	N/A	Dunja Knapp (CRTD)	PCR product
	PGK-Hygromycin	hSpCas9n-2A-Puro	N/A	Shahryar Khattak (CRTD)	PCR product
	3' GCG HA	genomic PCR fragment	this work	this work	PCR product
	pDTA-TK (plasmid backbone)	modified pDTA-TK*	this work	this work	NotI linearised
	5' MAFA HA	hMAFA 5pHA	this work	custom made, ThermoFisher	restriction fragment
MAFA ^{mCHERRY}	T2A-mCHERRY	pGEMT_EGFPbait-T2A-Cherry-3xNLS	N/A	Dunja Knapp (CRTD)	PCR product
	PGK-Hygromycin	E2-Crimson-IRES-Puro_PGK-Hygro	N/A	Shahryar Khattak (CRTD)	PCR product
	3' MAFA HA	hMAFA 5pHA	this work	custom made, ThermoFisher	restriction fragment
	pDTA-TK (plasmid backbone)	modified pDTA-TK**	this work	this work	NotI linearised
	5' INS HA	hINS 5pHA	this work	this work	PCR product
INS ^{GCaMP6}	GCaMP6	pGP-CMV-GCaMP6s	Chen et al. 2013	Addgene 40753	PCR product
	PGK-Neomycin	PGKneoF2L2DTA	Hoch and Soriano 2006	Addgene 13445.	PCR product
	3' INS HA	hINS 3pHA	this work	this work	PCR product

* The bGHpolyA from PGKneoF2L2DTA has substituted the SV40polyA of pDTA-TK as a SbfI / KpnI restriction fragment
** The βglob polyA from pGEMT_EGFPbait-T2A-Cherry-3xNLS has substituted the SV40polyA of pDTA-TK using the ligase chain reaction

Table S1 Generation and / or origin of the DNA fragments used for the Gibson assembly of the targeting constructs

Allele	CRISPR/Cas9 variant	sg RNA	Sequence	position
INS ^{eGFP}	nickase	hINS sgRNA 1<br>hINS sgRNA 2	GCTGGTAGAGGGAGCAGATGC (R)<br>GTGCAACTAGACGCAGCCCGC (F)	before STOP<br>overlapping STOP
GCG ^{mCHERRY}	wild type	hGCG sgRNA 1<br>hGCG sgRNA 3	AAACATCCCACGTGGCTAGC (R)<br>AACATTTCAAACATCCCACG (R)	before STOP<br>before STOP
MAFA ^{mCHERRY}	wild type	hMAFA sgRNA 1<br>hMAFA sgRNA 3	CGACTTCTTCCTGTAGGCGC (F)<br>CACGGCCGACTTCTTCCTGT (F)	overlapping STOP<br>overlapping STOP
INS ^{GCaMP6}	wild type	INS sgRNA 1<br>INS sgRNA 2	GCTGGTAGAGGGAGCAGATGC (R)<br>GTGCAACTAGACGCAGCCCGC (F)	before STOP<br>overlapping STOP

Table S2 CRISPR Cas9 variant, sequence of the revers (R) or forward (F) sg RNAs used and their position relative to the STOP codon

Allele	PCR product	Oligo name	Oligo sequence	Size
INS ^{eGFP}	5' HA + junctions	hINS F3	CAGGACAGGCTGCATCAGAA	2082
		eGFP R	ACGAACTCCAGCAGGACCAT	
	3' HA + junctions	eGFP F	CATCGACTTCAAGGAGGACGG	2353
		hINS R3	TTGTTGGCCATCAGGGTCAG	
GCG ^{mCHERRY}	5' HA + junctions	hGCG F	AGCCTAGAAGATTGAAAAGGGCA	1373
		mCherry R	CAAGTAGTCGGGGATGTCGG	
	3' HA + junctions	mCherry F	TTCGAGATCGAGGGCGAGG	3892/2015
		hGCG R	ACCCTATGGGAGCACTATAGAGATAGAGAAAG	
MAFA ^{mCHERRY}	5' HA + junctions	hMAFA F	TCTGAGTTGCCATGGGGATAAGCAAATGA	1840
		mCherry R	CAAGTAGTCGGGGATGTCGG	
	3' HA + junctions	mCherry F	TTCGAGATCGAGGGCGAGG	4200/2323
		hMAFA R	AGGCTGCGGTGACTTGGATCTGAACA	
INS ^{GCaMP6}	5' HA + junctions	hINS F3	CAGGACAGGCTGCATCAGAA	2113
		eGFP R2	CTTGTAGTTGCCGTCGTCCT	
	3' HA + junctions	eGFP F2	ATGGTCCTGCTGGAGTTCGT	4665/2986
		hINS R3	TTGTTGGCCATCAGGGTCAG	

Table S3 Genotyping and validation strategy of the targeted alleles. The amplified fragments were then Sanger sequenced.

LINE	CLONES
INS ^{eGFP}	# 4-1
	# 32-1
	# 36-1
INS ^{eGFP} / *GCG* ^{mCHERRY}	# 24-8
	# 59-6
INS ^{eGFP} / *MAFA* ^{mCHERRY}	# 59-1
	# 63-1
INS ^{GCaMP6}	# 9-1
	# 96-2

Table S4 List of lines and corresponding expanded clones. Used clones are highlighted

Stage	Duration (days)	Basal media	Supplements
**S1 (DE)**	**3**	MCDB131 (Life Technologies, 10372-019)<br>2.5mM Glucose (Sigma, G8769)<br>1x Glutmax (Life Technologies, 35050038)<br>1.5 g/l NaHCO3 (Sigma-Aldrich, S6297)<br>0.5 % BSA (Serva, 11945)<br>1x Pen/Strep (Life technologies, 15140-122)	100 ng/ml Activin A (Peprotech, 120-14P) (all three days)<br>3.0 µM of CHIR (Tocris, SML1046) (1st day)<br>0.3 µM of CHIR (Tocris, SML1046) (2nd day)
**S2 (PGT)**	**2**		50 ng/ml FGF7 (Peprotech, 100-19)<br>0.25 mM Vitamin C (Tocris, 4055)<br>IWP-2 (Tocris, 3533)
**S3 (PF)**	**2**	MCDB131 (Life Technologies, 10372-019)<br>4.5 mM Glucose (Sigma, G8769)<br>1x Glutmax (Life Technologies (35050038)<br>1.5 g/l NaHCO3 (Sigma-Aldrich S6297)<br>2% BSA (Serva, 11945)<br>1x Pen/Strep (Life technologies, 15140-122)<br>0.5% IST-X (Life technologies, 51500056)	50 ng/ml FGF7 (Peprotech, 100-19)<br>0.25 mM Vitamin C (Tocris, 4055)<br>0.25 µM SANT1 (Sigma, S4572)<br>100 nM LDN (Sigma, SML0559)<br>1 µM Retinoic Acid (Sigma, R2625)<br>200 nM TPB (Merck, 565740)
**S4 (PP)**	**3**		0.25 mM Vitamin C (Tocris, 4055)<br>2 ng/ml FGF7 (Peprotech, 100-19)<br>0.25 µM SANT1 (Sigma, S4572)<br>0.1 µM Retinoic Acid (Sigma, R2625)<br>200 nM LDN (Sigma, SML0559)<br>100 nM TPB (Merck, 565740)
**S5 (PEP)**	**4**	MCDB131 (Life Technologies, 10372-019)<br>14.5 mM Glucose (Sigma, G8769<br>1x Glutmax (Life Technologies, 35050038)<br>1.5 g/l NaHCO3 (Sigma-Aldrich, S6297)<br>2% BSA (Serva, 11945)<br>1x Pen/Strep (Life Technologies, 15140-122)<br>0.5 x IST-X (Life Technologies, 51500056)<br>1.8u/ml Heparin (Sigma, 2106-10VL)	0.25 µM SANT1 (Sigma, S4572)<br>0.05 µM Retinoic Acid (Sigma, R2625)<br>100 nM LDN (Sigma, SML0559)<br>10 µM ALK5i (Milltenybiotech, 130117340)<br>1 µM T3 (Sigma, T6397)<br>10 µM ZnSO4 (Sigma, Z0251)
**S6**	**7**		100 nM LDN (Sigma, SML0559)<br>10 nM XX (Millipore, 565789)<br>10 µM ALK5i (Millteny Biotech, 130117340)<br>1 µM T3 (Sigma, T6397)<br>10 µM ZnSO4 (Sigma, Z0251)
**S7**	**10-14**	MCDB131 (Life Technologies, 10372-019)<br>1x Glutmax (Life Technologies, 35050038)<br>1.5 g/l NaHCO3 (Sigma-Aldrich, S6297)<br>0.5 mM Sodium Pyruvate (Lonza BE13-115E)<br>2% BSA (Serva, 11945)<br>1x Pen/Strep (Life Technologies, 15140-122)<br>0.5 x IST-X (Life Technologies, 51500056)<br>1.8u/ml Heparin (Sigma, 2106-10VL)	1mM N-Cyst (Sigma, A9165)<br>2 µM R428 (Selleckchem, S2841)<br>10 µM ALK5i (Millteny Biotech, 130117340)<br>1 µM T3 (Sigma, T6397)<br>10 µM ZnSO4 (Sigma, Z0251)<br>10 µM Trolox (Millipore, 648471)

Table S5 Basal media and supplements for S1-S7

Antigen	Species	Supplier, cat no	Application	Dilution
OCT4	Mouse	Santa Cruz, sc-5279	IF/FC	1:100/1:200
SOX2	Rabbit	Abcam, ab97959	IF/FC	1:200/1:100
SSEA4	Mouse	DSHB, MC-813-70	IF	1:80
FOXA2	Rabbit	Merck, 07-633	IF/FC	1:200
SOX17	Mouse	R&D, MAB1924	IF/FC	1:50
NKX6.1	Mouse	DSHB (F55A10)	IF/FC	1:500/1:250
PDX1	Goat	R&D, AF2419	IF/FC	1:40
SOX9	Rabbit	Merck, AB5535	IF/FC	1:1000
GCG	Mouse	Sigma, G2654	IF	1:500
INS	Guinea pig	Abam, ab7842	IF	1:500
MAFA	Rabbit	Abcam, ab264418	IF	1:300
GFP	Chicken	Abcam, ab13970	IF	1:2000
mCherry	Rat	Chromotek, 5f8	IF	1:1000

Table S6 List of primary antibodies for immunofluorescense (IF) and flow cytometry (FC)

Antigen	Fluorophore	Species	Supplier, cat no	Dilution
Goat IgG	Alexa 647	Donkey	Invitrogen, A21447	1:500
Guinea pig IgG	Alexa 647	Goat	Abcam, ab150187	1:500
Mouse IgG	Alexa 568	Donkey	Invitrogen, A10037	1:500
Mouse IgG	Alexa 568	Goat	Invitrogen, A11004	1:500
Chicken IgY	Alexa 488	Goat	Abcam, ab150169	1:500
Rabbit IgG	Alexa 488	Donkey	Invitrogen, A21206	1:500
Rabbit IgG	Alexa 488	Goat	Invitrogen, A11070	1:500
Rat IgG	Alexa 568	Goat	Invitrogen, A11077	1:500

Table S7 List of secondary antibodies

Stage	Gene and primer direction	Sequence
Housekeeping	*TBP F*	TATCGTGGTCACACTTGTTGAG
	TBP R	ACCATCCTCTGAGACCGTTTT
PP	*PDX1 F*	GGAGAAAGATGGACCCCTGG
	PDX1 R	CAGCCTCTACCTCGGAACAG
	SOX9 F	CGGAGGAAGTCGGTGAAG
	SOX9 R	CCTTGAAGATGGCGTTGG
	NKX6.1 F	ATTCGTTGGGGATGACAGAG
	NKX6.1 R	CGAGTCCTGCTTCTTCTTGG
	PTF1A F	TTCACCGACCAGTCTTCACG
	PTF1A R	GTGGCTAAGGAACTCCACCT
	AFP F	GCAGCAGTCTGAATGTCCGTAC
	AFP R	TGCCCAGTTTGTTCAAGAAGC
	CDX2 F	GCTGGAGAAGGAGTTTCACTACAGT
	CDX2 R	AACCAGATTTTAACCTGCCTCTCA
PEP	*PDX1 F*	GGAGAAAGATGGACCCCTGG
	PDX1 R	CAGCCTCTACCTCGGAACAG
	SOX9 F	CGGAGGAAGTCGGTGAAG
	SOX9 R	CCTTGAAGATGGCGTTGG
	NKX6.1 F	ATTCGTTGGGGATGACAGAG
	NKX6.1 R	CGAGTCCTGCTTCTTCTTGG
	NEUROG3 F	TGGGTGCTAAGGGTAAGGGA
	NEUROG3 R	CAGCCAGGGAGAAGCAGAAG
	NEUROD1 F	AGGCAGCCCTTTGGGTACTA
	NEUROD1 F	TTGATCCCCTGTTTCTTCCA
	AFP F	GCAGCAGTCTGAATGTCCGTAC
	AFP R	TGCCCAGTTTGTTCAAGAAGC
	CDX2 F	GCTGGAGAAGGAGTTTCACTACAGT
	CDX2 R	AACCAGATTTTAACCTGCCTCTCA
S7	*PDX1 F*	GGAGAAAGATGGACCCCTGG
	PDX1 R	CAGCCTCTACCTCGGAACAG
	NKX6.1 F	ATTCGTTGGGGATGACAGAG
	NKX6.1 R	CGAGTCCTGCTTCTTCTTGG
	GCG F	GAGACATGCTGAAGGGACCT
	GCG R	CTTCCTCGGCCTTTCACCAG
	SST F	AGCTGCTGTCTGAACCCAAC
	SST R	GCTCAAGCCTCATTTCATCC
	GK F	AGCGTGAAGACCAAACACCA
	GK R	ATGCTTGTCCAGGAAGTCGG
	MAFA F *	GCGGAGAACGGTGATTTCTA
	MAFA R *	GAAGGTGGGAACGGAGAAC
	INS F	AGATCACTGTCCTTCTGCCA
	INS R	CGCACAGGTGTTGGTTCA
	PC1/3 F	TACAAGCACAGAGACGACCG
	PC1/3 R	CGCAGGGTAAGGAAGAAGCA
	PAX4 F	GTGATTGCTCCCTCCTGTGT
	PAX4 R	GTGAGAAGTGGGTGGGTGTT
	CK19 F	TGCCTTGGAAGACACACTGG
	CK19 R	CTGGGCTTCAATACCGCTGA
	SLC30A8 (ZnT8) F	GGCCGTCATGGAGTTTCTT
	SLC30A8 (ZnT8) R	CACCGGTTTCTGTTGGAGTT

Table S8 List of forward (F) and reverse (R) primers used for qPCR

* because of the very high GC content of the MAFA coding region the reverse primer had to be designed against the 3' UTR. As a consequence, this pair of primers does not recognise the targeted allele
